# The role of the brown bear *Ursus arctos* as a legitimate megafaunal seed disperser

**DOI:** 10.1038/s41598-020-80440-9

**Published:** 2021-01-14

**Authors:** Alberto García-Rodríguez, Jörg Albrecht, Sylwia Szczutkowska, Alfredo Valido, Nina Farwig, Nuria Selva

**Affiliations:** 1 Institute of Nature Conservation, Polish Academy of Sciences, Al. Adama Mickiewicza 33, 31-120 Kraków, Poland; 2 Senckenberg Biodiversity and Climate Research Centre (Sbik-F), Senckenberganlage 25, 60325 Frankfurt am Main, Germany; 3 Workshop for All Beings Association, Jasna 17, 43-360 Bystra, Poland; 4 Island Ecology and Evolution Research Group, Instituto de Productos Naturales Y Agrobiología (IPNA-CSIC), C/Astrofísico Francisco Sánchez 3, 38206 La Laguna, Tenerife Spain; 5 Conservation Ecology, Faculty of Biology, University of Marburg, Karl-von-Frisch-Straße 8, 35043 Marburg, Germany

**Keywords:** Ecology, Ecosystem services, Seed distribution

## Abstract

Megafaunal frugivores can consume large amounts of fruits whose seeds may be dispersed over long distances, thus, affecting plant regeneration processes and ecosystem functioning. We investigated the role of brown bears (*Ursus arctos*) as legitimate megafaunal seed dispersers. We assessed the quantity component of seed dispersal by brown bears across its entire distribution based on information about both the relative frequency of occurrence and species composition of fleshy fruits in the diet of brown bears extracted from the literature. We assessed the quality component of seed dispersal based on germination experiments for 11 fleshy-fruited plant species common in temperate and boreal regions and frequently eaten by brown bears. Across its distribution, fleshy fruits, on average, represented 24% of the bear food items and 26% of the total volume consumed. Brown bears consumed seeds from at least 101 fleshy-fruited plant species belonging to 24 families and 42 genera, of which *Rubus* (Rosaceae) and *Vaccinium* (Ericaceae) were most commonly eaten. Brown bears inhabiting Mediterranean forests relied the most on fleshy fruits and consumed the largest number of species per study area. Seeds ingested by bears germinated at higher percentages than those from whole fruits, and at similar percentages than manually depulped seeds. We conclude that brown bears are legitimate seed dispersers as they consume large quantities of seeds that remain viable after gut passage. The decline of these megafaunal frugivores may compromise seed dispersal services and plant regeneration processes.

## Introduction

Seed dispersal (i.e. the movement of seeds away from the parent plants) is essential for plant recruitment, colonization of habitats, gene flow among populations and plant community dynamics^1,2^. Across different biomes, a large proportion of vascular plant species depends on frugivorous animals for the dispersal of their seeds^3^. The spatial distribution of seeds dispersed by frugivores is strongly affected by animal species body size and mobility^4–6^. Large frugivore species seem to be particularly important in connecting plant populations by increasing gene flow via dispersed seeds^7–9^.

Beyond differences in their mobility across landscapes, frugivores also vary in their seed dispersal effectiveness, which depends on the qualitative and quantitative contribution to seed dispersal services^10,11^. The quantity component is defined as the number of seeds dispersed, which is determined by both the number of interactions between a disperser agent and a fruiting plant species and the number of seeds removed by a disperser per interaction. The quality component is traditionally defined as the probability that a dispersed seed will germinate, survive and grow to an adult plant, which is determined by the combined effects of fruit handling, gut passage treatment and seed deposition in suitable microhabitats^10^. Legitimate seed dispersers are usually defined as true mutualist agents that combine a high quality and quantity of seed dispersal and, thus, strongly impact regeneration processes and population dynamics of the dispersed plant species^12^.

Traditionally, most studies about frugivory and seed dispersal have been focused on birds and small- to medium-sized mammals^13,14^. In the last two decades, an increasing number of studies about seed dispersal by extant large frugivores from tropical areas have been published^14^. However, the information about the role of large frugivores as seed dispersers in temperate and northern regions is still limited. Megafaunal species are currently defined as comparatively large animal species that have strong effects on ecosystems, present distinctive functional traits and habitat requirements and have escaped most-non anthropogenic predation when adults^15^. Megafaunal frugivores are considered quantitatively and qualitatively pivotal seed dispersers, particularly because they can transport many seeds over long distances^16^. The increased chance of long-distance dispersal events by these animals facilitates the colonization and re-colonization of new and former habitats by the plant species they consume, enhancing genetic diversity and reducing parent-sibling competition^12^. In addition, megafaunal frugivores have the potential to consume many different fleshy-fruited plants, including species with both small and big seeds^17^. The selective loss of large-bodied animals during the last centuries^18^, particularly megafauna, can strongly impair the dispersal of large-seeded plant species, which may have serious consequences for their recruitment, population structure, genetic diversity and evolutionary trajectories^6,9,16,19^. Therefore, complete information about the role of extant megafauna for seed dispersal and plant regeneration processes is highly valuable.

**Figure 1:**
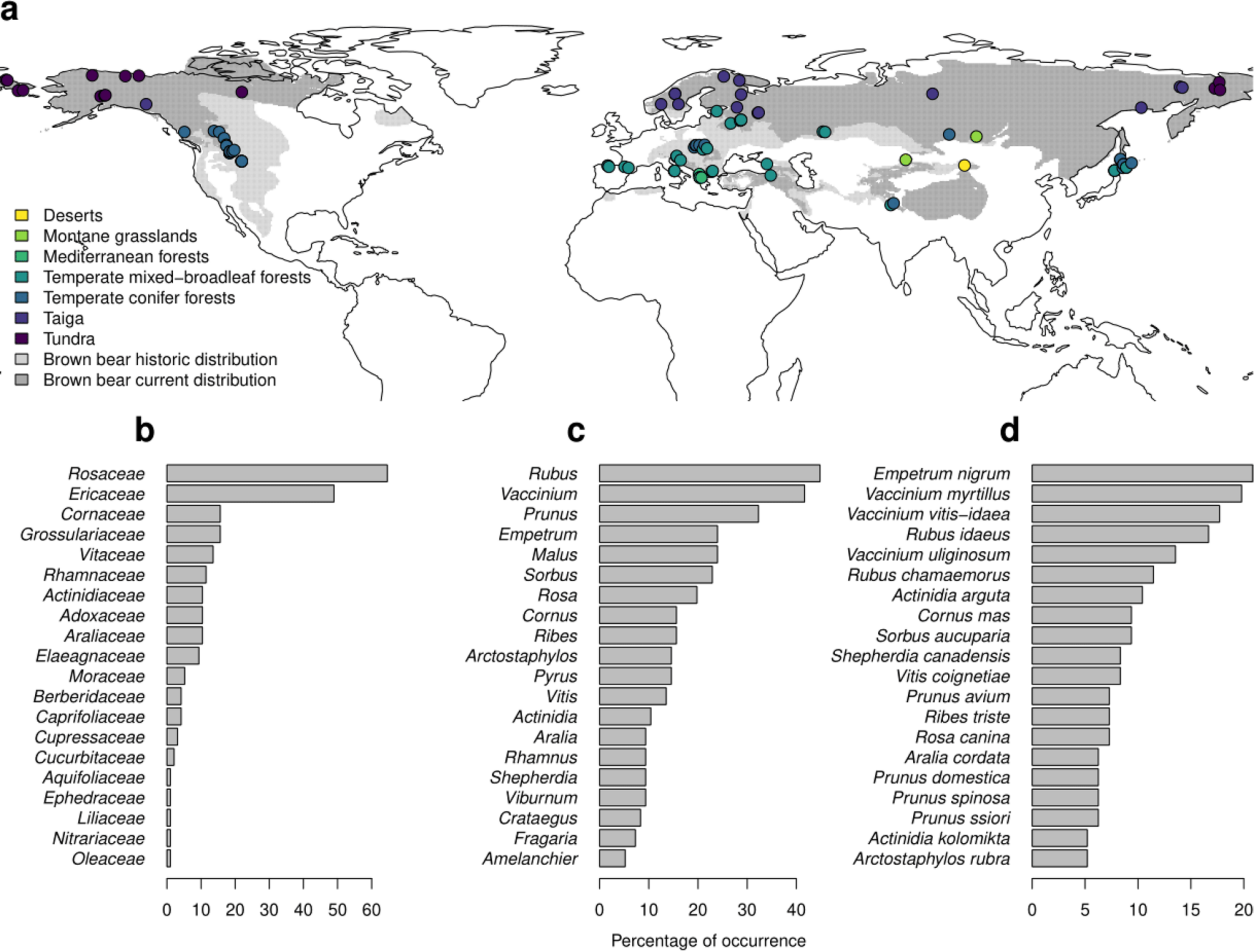
(**a**) Map showing the study areas (n = 96, dots) from which data on brown bear diet were gathered. Brown bear distribution is shown in grey. The dot colors represent the biomes in which each study area is located. The percentages of study areas with presence of different families (**b**), genera (**c**) and species (**d**) of fleshy fruits are shown in the lower panels. Only the 20 most common taxa of each taxonomic rank are shown. Study areas are listed in Appendix S6 and the complete list of taxa recorded in the brown bear diet worldwide is presented in Appendix S1. The map in (**a**) was created with the R statistical environment (version 3.4.0)^79^, using the package *rworldmap*^80^. Historical and present brown bear distributions were extracted from the IUCN website^81^.

The brown bear *Ursus arctos* (Order: Carnivora, Family: Ursidae) is one of the world’s most widely distributed terrestrial mammals and the largest living terrestrial carnivore; it inhabits a broad variety of biomes, from tundra to deserts (Fig. 1). Its heavy body mass and large habitat requirements^20^, together with a distinctive biology (e.g. late first reproduction in females and longer maternal care when compared to other carnivore species^21^) and the lack of non-human natural predators at adult stages make the species a good representative of megafauna inhabiting northern latitudes. The brown bear is an omnivore with an important share of fleshy-fruited plants in its diet. Its trophic niche is very flexible and strongly related to environmental conditions, with populations in warm and highly productive environments being almost completely herbivorous, whereas populations in cold, unproductive environments are more carnivorous^22^. Previous studies have shown that resource availability influences the age of first reproduction, litter size, population density, home range size and habitat selection in brown bear populations^23–28^. Besides these more general effects of resource availability, the abundance of fruit resources is known to strongly affect female breeding success in some brown bear populations^29^. This indicates that fleshy fruits are a key food resource for the species. However, our understanding of the reciprocal effect of the brown bear on the dispersal and regeneration of its food plants remains incomplete; particularly the assessment of the seed dispersal service provided by the species.

Our main goal was to assess the role of the brown bear as a legitimate megafaunal seed disperser across its entire distribution range, addressing both the quantity and quality components of the seed dispersal effectiveness provided by the species. We specifically aimed to (1) identify all fleshy-fruited plant species eaten by brown bears worldwide, (2) evaluate the contribution of fleshy fruits in brown bear diet across biomes, and (3) determine the effects of ingestion by bears on the proportion and speed of germination in selected fleshy-fruited plant species commonly eaten by brown bears. We also explored the factors related to brown bear biology and ecology that may influence its seed dispersal effectiveness.

## Results

### Species richness and quantity of fleshy fruits consumed by brown bears

We found that brown bears consumed fleshy fruits in the seven biomes where they were present. At least 101 fleshy-fruited plant species belonging to 42 genera and 24 families were eaten by brown bears across the 96 study areas (Table 1, Fig. 1, Appendix S1). Fruits from Rosaceae and Ericaceae families were the most frequently consumed, being recorded in 65% and 49% of the study areas, respectively. The genus most commonly consumed by bears was *Rubus*, appearing in 45% of the studied areas, followed by *Vaccinium* (42%) and *Prunus* (32%, Fig. 1). Regarding species, *Empetrum nigrum* (present in the diet in 21% of the study areas), *V. myrtillus* (20%), *V. vitis-idaea* (18%) and *Rubus idaeus* (17%) were the most common fleshy-fruited plants eaten (Fig. 1). On average, brown bears consumed almost five fleshy-fruited plant species per study area, and fleshy fruits represented 24% of the consumed food items (relative frequency of occurrence, Table 1) and 26% of the total volume of all bear foods (n = 46 study areas). At the study area level, the percentage of volume of fleshy fruits in bear diet was highly correlated with the relative frequency of occurrence (Pearson's product-moment r = 0.82, n = 46 study areas).

**Table 1:** Average values (mean ± SD) among study areas of the number of taxa (species and genera) and relative frequency of occurrence (number of occurrences of fleshy-fruited plant species divided by the total number of occurrences of all food items consumed) of fleshy fruits eaten by brown bears in each biome within brown bear distribution range and in the entire range. The total number of studies and samples examined is also indicated for each biome. The same letter (a, b, c) after the scores of the relative frequency of occurrence in different biomes indicate statistical differences between these biomes (e.g. letter “a” represents statistical differences in the frequency of occurrence of fleshy fruits in brown bears diet between boreal forests and taiga and temperate coniferous forests).

Biome	No. species/study area (mean ± SD)	No. genera/study area (mean ± SD)	Relative frequency of occurrence (mean ± SD)	No. study areas	No. samples
Tundra	3.43 ± 1.22	3.00 ± 1.11	0.25 ± 0.12	14	2696
Boreal forests and taiga	4.87 ± 3.40	3.40 ± 2.06	0.27 ± 0.14^a^	15	4431
Temperate coniferous forests	4.40 ± 3.80	4.03 ± 3.47	0.16 ± 0.08^abc^	30	17,272
Temperate mixed and broadleaf forests	5.53 ± 4.15	5.20 ± 4.04	0.26 ± 0.13^b^	30	10,272
Montane grasslands and shrublands	5.00 ± 5.61	5.00 ± 5.66	0.24 ± 0.18	2	1779
Mediterranean forests, woodlands and scrubs	7.25 ± 3.31	7.00 ± 3.37	0.45 ± 0.21^c^	4	1134
Deserts and xeric shrublands	1.00 ± NA	1.00 ± NA	NA	1	365
Average/total	4.78 ± 3.63	4.26 ± 3.35	0.24 ± 0.14	96	37,949

**Figure 2:**
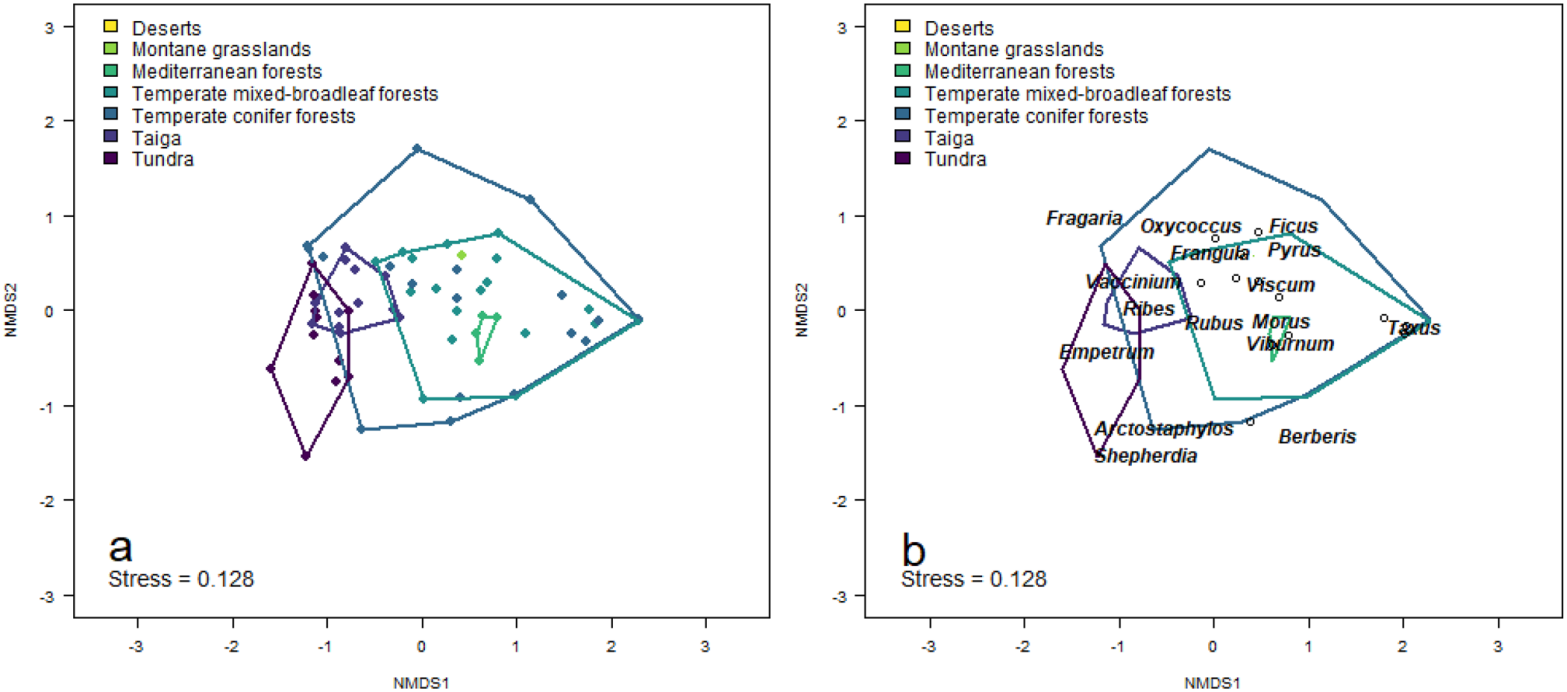
Nonmetric multidimensional scaling ordination plots illustrating differences in the contribution of fleshy-fruited plant genera consumed by brown bears across biomes. These differences are based on the relative frequency of occurrence of each genus in brown bear diet in 66 study areas. The location within the ordination plot of the study areas from which data were gathered (**a**) and the fleshy-fruited plant genera eaten by brown bears (**b**) are shown. *Shepherdia* and *Empetrum* are consumed by brown bears mostly in boreal biomes (i.e. tundra and taiga; left part in **b**), while *Vaccinium* and *Ribes* are eaten in both boreal and temperate regions (mid-left in **b**). The majority of the genera consumed by brown bears are found in temperate regions (mid part in **b**), with *Viburnum*, *Prunus* and *Cornus* being mostly eaten by brown bears inhabiting Mediterranean regions.

The average number of fleshy-fruited taxa and the relative frequency of occurrence of fleshy fruits in brown bear’s diet were the highest in Mediterranean areas, whereas intermediate values were observed in temperate and boreal regions (Table 1, Fig. 2, Appendix S2). The lowest numbers of fleshy-fruited plant taxa were consumed by brown bears in deserts and in the tundra, but also diet studies in these biomes were scarce (Table 1, Appendix S2). The ordination plot (Fig. 2) indicated that genera belonging to the Ericaceae family (e.g., *Vaccinium*, *Empetrum* and *Arctostaphylos*) were mainly associated with boreal biomes, i.e. tundra and taiga, whereas genera belonging to the Rosaceae (e.g. *Malus*, *Pyrus*, *Rosa*, *Rubus*) and Rhamnaceae (e.g. *Rhamnus* and *Frangula*) families were mainly associated with temperate biomes. Species from *Prunus*, *Viburnum* and *Cornus* genera were associated with both Mediterranean and temperate biomes. We found statistical differences in the relative frequency of occurrence of each fleshy-fruited plant genera eaten by brown bears among biomes (results from permanova test based on Bray–Curtis distances and 999 permutations: R^2^ = 0.28; *p* value = 0.001).

The mean number of seeds per brown bear scat found in the Bieszczady Mountains varied among fleshy-fruited plant species, ranging from 6344 seeds in the case of *Rubus fruticosa* to only two seeds in the case of *Frangula alnus* (Table 2).

**Table 2:** Characterization of some of the quality and quantity components of brown bear seed dispersal for 11 fleshy-fruited plant species commonly eaten by brown bears in Eurasian temperate forests. The table shows the life form (tree, shrub or both), fruit type (berry, drupe, polydrupe or pome), number of seeds per fruit (average and range), average weight of 1,000 seeds (gr—information extracted from the Kew Royal Botanical Garden), average number of seeds found per bear scat (n = 100 scats containing fleshy fruits) and the percentage of seeds that remain intact after bear ingestion. The results of the germination experiment are also shown and include the percentage of seeds germinated at the end of the 2-year experiment and the mean germination time during the first year of germination (in number of days elapsed since April 1st) for the three treatments (Brown bear-seeds ingested by bears and recovered from the scats, Depulped-manually depulped fruits, and Whole fruits-seeds embedded within the whole fruit). * Asterisks mark species that only germinated during the second year of germination.

Species	Life form	Fruit type	No. seeds per fruit (range)	Weight 1000 seeds (gr)	No. seeds per scat (% intact seeds)	Germination treatments					
						Brown bear		Depulped		Whole fruits	
						Seeds germinated (%)	Mean germination time (days)	Seeds germinated (%)	Mean germination time (days)	Seeds germinated (%)	Mean germination time (days)
*Rosa sp.*	Shrub	Pome	25 (16–53)	16.0	4 (100%)	54.4	21.45	52.4	25.04	7.61	20.30
*Frangula alnus*	Tree/shrub	Drupe	2.5 (2–4)	20.6	2 (100%)	71.8	26.95	61.2	26.26	37.6	29.22
*Vaccinium myrtillus*	Shrub	Berry	52 (15–86)	0.3	2,190 (98.9%)	22.4	49.23	30.0	49.51	0.46	49.17
*Rubus fruticosa*	Shrub	Polydrupe	29 (7–44)	2.23	6,344 (98.7%)	60.8	49.88	45.6	58.21	20.3	39.42
*Sambucus nigra*	Tree/shrub	Drupe	3 (2–4)	12.0	136 (97.7%)	77.8	15.21	65.2	22.07	36.3	12.99
*Sorbus aucuparia*	Tree	Pome	2.2 (1–4)	7.0	242 (93.3%)	69.8	12.10	46.0	14.22	46.8	NA
*Prunus spinosa*	Shrub	Drupe	1 (1–1)	175	224 (88.7%)	79.8	19.81	76.0	15.70	46.0	27.50
*Prunus avium*	Tree	Drupe	1 (1–1)	183	875 (62.3%)	20.6	14.86	27.2	14.01	33.0	8.67
*Malus sp.*	Tree	Pome	4.2 (2–8)	12.4	74 (48.8%)	88.8	9.75	69.8	10.61	13.8	16.40
Viburnum opulus*	Tree/shrub	Drupe	1 (1–1)	33.3	–	49.6	–	55.4	–	65.0	–
Crataegus monogyna*	Tree	Pome	1 (1–1)	98.0	–	24.8	–	25.8	–	20.0	–

### Quality of the seeds dispersed by brown bears

After assessing the taxonomic composition and the frequency of occurrence of fleshy-fruits in brown bear diet across the 96 study areas and the number of seeds per scat found in the Bieszczady Mountains (quantity component), we identified in detail the consequences of gut passage for the intactness and germination of seeds recovered from brown bear scats. Across the nine plant species recorded in bear scats in the Bieszczady Mountains, on average 88% of the seeds remained undamaged after bear ingestion (Table 2). In six out of nine species more than 93% of the seeds remained intact after bear ingestion, whereas in *Prunus avium* and *Malus sp.* the percentage was lower, with 62% and 49% of the seeds undamaged after gut passage, respectively (Table 2). We found that the seed weight was not related to the percentage of seeds undamaged after bear gut passage (Spearman’s rank correlation: rho = − 0.44, n = 9).

**Figure 3:**
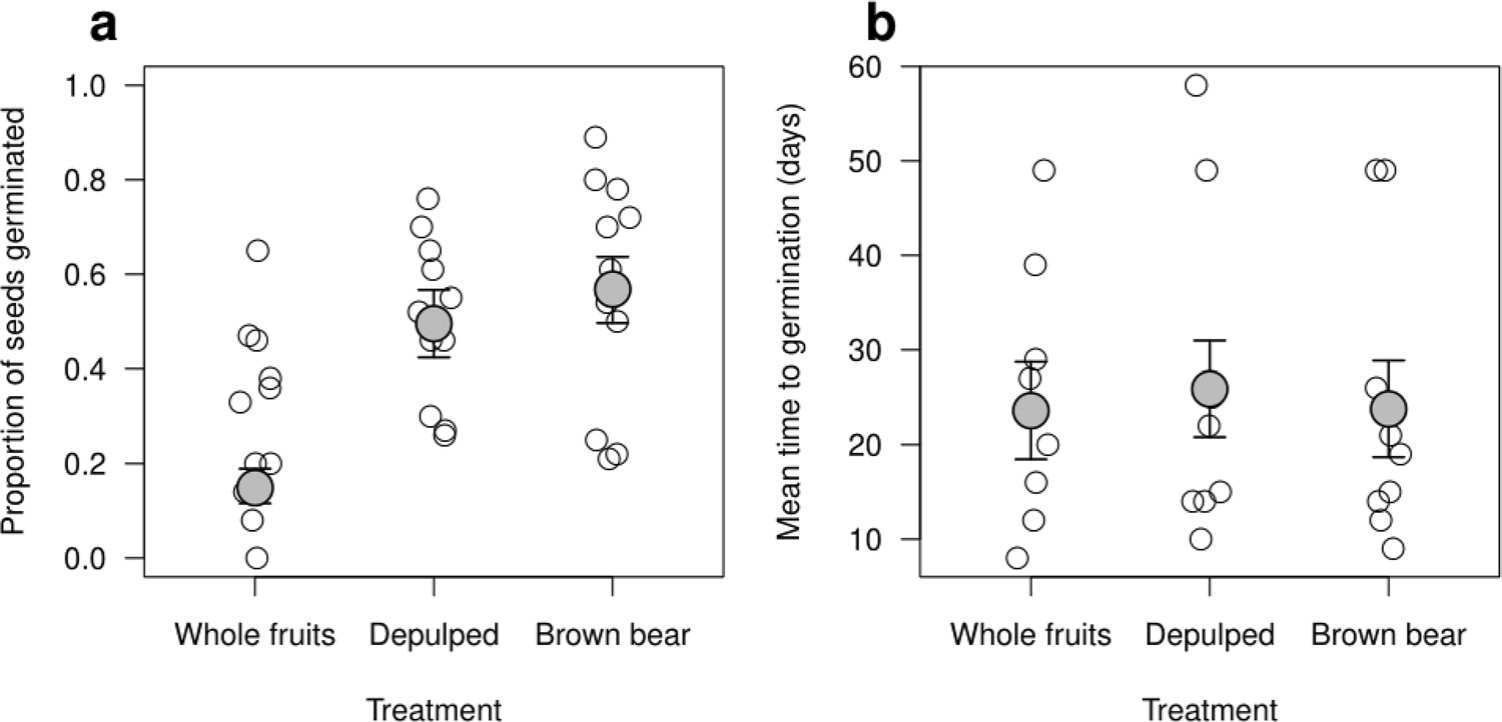
(**a**) Mean proportion of seeds germinated at the end of the 2-year germination experiment and (**b**) mean germination times during the first year of germination (number of days elapsed since April 1st) for 11 fleshy-fruited plant species in relation to three germination treatments (Brown bear-seeds ingested by brown bears and recovered from the scats, Depulped-manually depulped fruits and, Whole fruits-seeds embedded within the whole fruit). Grey dots and black arrows represent the predicted mean values and the standard errors, respectively. Empty dots represent the actual observed values for each fleshy-fruited plant species sown. Fleshy-fruited plant species are listed in Table 2.

In general, seeds ingested by bears and manually depulped had higher percentages of germination than seeds sowed within the pulp (Table 2, Fig. 3, Appendix S3). Seeds ingested by brown bears germinated better than seeds manually depulped and whole fruits in seven out of the eleven fleshy-fruited plant species (Table 2). Germination percentages after bear ingestion were higher than 50% for seven species, whereas in the case of depulped and whole fruit treatments, six and one species exceeded this germination percentage, respectively (Table 2). Seeds from *V. myrtillus* and *F. alnus* germinated only during the first year, while *V. opulus* germinated exclusively in the second year (Appendix S4 and S5). We did not find any influence of the treatment on the mean germination times (Fig. 3, Appendix S3).

## Discussion

Our study provides a first comprehensive assessment of the role of brown bears as legitimate seed dispersers across their distribution range. We have shown that frugivory in the brown bear is not just a locally restricted phenomenon, but that fleshy fruits represent a major food resource for the species across its entire distribution. Across its geographic range the brown bear consumes fruits of more than 100 plant species belonging to 42 genera and 24 families, whose seeds remain mostly intact after bear ingestion and germinate better than when embedded within the pulp. The quantity and quality of seed dispersal services provided by brown bears highlight that this megafaunal species is a legitimate seed disperser and may have a substantial impact on plant regeneration services in all the biomes where the species is present. Therefore, brown bears must be recognised as one of the few extant and relevant legitimate megafaunal seed dispersers inhabiting non-tropical areas.

Our literature review revealed that frugivory by brown bears is prevalent across the species entire geographic range, and represents on average a quarter of the total volume and of the number of food items eaten during an entire year. As fleshy fruits are not available all year around in northern regions, these figures would probably be much higher if only the fruiting period would be considered. Previous studies analysing global dietary patterns of other frugivorous mammals sympatric with brown bears, such as red foxes *Vulpes vulpes* and pine martens *Martes martes*, has revealed that these species also track fruit availability and change their dietary patterns along the year, feeding primarily on small vertebrates during winter, spring and early summer. Plants are less important in the diets of these mesocarnivores, being consumed mostly during late summer and autumn, and, on average, not exceeding 20% of the annual volume^30,31^. Large herbivores sympatric with brown bears and considered as megafauna, such as the European bison *Bison bonasus* or the red deer *Cervus elaphus*, also disperse viable seeds from more than a hundred plant species, whereas others like the moose *Alces alces* are less efficient dispersers^32,33^. However, wild ungulates feed primarily on herbs and leaves, with fruits representing less than 5% of the total volume of their diet^34,35^. Additionally, the germination success of seeds defecated by ungulates is usually below 10%^36^. Therefore, in comparison with sympatric megafauna, brown bears are among the most effective megafaunal seed dispersers in their distribution range, at least in areas where other ursid species are absent.

The importance of fleshy fruits in brown bear diet can be partly explained by the annual cycle of the brown bear. As a hibernator, the species is adapted to seasonal climates with prolonged periods of energetic bottlenecks^37^. Successful hibernation depends on the energy reserves at the onset of the denning period, which are crucial to survive the winter. To meet the energetic demands during hibernation, brown bears maximize their energy uptake during a period of hyperphagia, in which they feed intensively on fleshy fruits and mast to build up body fat before den entry^25,29^. Therefore, fleshy fruits represent a key food resource for the species, which can affect important aspects of brown bear biology such as habitat selection and breeding success^28,38^. Fleshy fruits contain an important proportion of hydrophobic lipids^39^, used to gain body fat for hibernation. Importantly, the ripening time of most fleshy fruits coincides with the hyperphagic period of highly food demand by bears, when they spend most of their time foraging. In that period, brown bears can consume up to a third of their body weight of fleshy fruits per day^25^.

Fleshy fruits are present in more than half of brown bears scats during late summer and early autumn and this applies to populations from different latitudes, including those from Northern Yukon, Hokkaido or the Pyrenees^40–42^. Other bear species such as the Asiatic black bear *U. thibetanus* and the American black bear *U. americanus* also feed intensively on fleshy fruits during the fruiting season and berries may represent more than the half of the total volume consumed^43,44^. An increased frugivory during late summer and early autumn is a common phenomenon in other carnivores inhabiting boreal and temperate regions such as red foxes or pine martens^32,33^*.* However, these species feed primarily on vertebrates during mid-summer and the share of fleshy fruits in the diet of these species is much lower than in the brown bear during this period.

The most common genera eaten by brown bears across the species range are *Rubus*, *Empetrum* and *Vaccinium*. These genera typically form dense vegetation layers at ground level with exceptionally high local fruit abundances. Thus, these plant genera are attractive resources because, once a fruiting patch has been detected, brown bears can easily harvest large amounts of fruits growing close to the ground by ‘browsing’ the local vegetation^25^. Fruits from four species of the Ericaceae familiy (*Empetrum nigrum, Vaccinium myrtillus, V. vitis-idaea* and *V. uliginosum*) are the most commonly eaten by brown bears in boreal regions. These few species dominate the ground layer in forests and meadows at northern latitudes, producing large amounts of nutritious and easily accessible fruits during late summer and autumn, when brown bears rely the most on fleshy fruits.

Up to our knowledge, comprehensive reviews on frugivory by given species at the global scale are scarce and the comparison between the number of fleshy-fruited plant species eaten by bears and other frugivores, including other ursids and megafauna species, is difficult to make, particularly in boreal and temperate regions. Megafaunal seed dispersers have been more studied in tropical areas, where more diverse fleshy fruits are highly available year-round. For instance, the Asian elephant *Elephas maximus* is known to disperse seeds of at least 122 species from 39 different families^45^, a number that is somehow comparable to the diversity of fleshy fruits consumed by brown bears, moreover considering that brown bears inhabit areas less rich in fleshy fruits than the tropics.

Brown bears are more frugivorous and consume the largest variety of fleshy-fruited plant species per study area in Mediterranean regions. These results support previous findings that brown bears inhabiting warmer and more productive areas rely more on fleshy fruits^22^. As brown bears are omnivores that feed opportunistically on many food resources, their diet may reflect the local availability and diversity of fruiting plant species in a given study area rather than strong dietary preferences. This is supported by the fact that Mediterranean high mountains scrublands, which are the areas where brown bears are still present in the Mediterranean basin, show very high fruit densities, only comparable to the exceptionally high fruit availability in tropical forests^3^. Brown bears consume the lowest number of fleshy-fruited plant species in cold and less productive areas, which may be also explained by the lower availability of these food resources.

We showed that a single brown bear scat may contain up to several thousand seeds, which supports a previous study conducted in Alaska where brown bears faeces containing up to 7,000 seeds of *Vaccinium* and 2,000 of *Ribes* species were recovered from the field^46^. This confirms that brown bears have the potential to disperse high quantities of seeds. However, the quantity component of the seed dispersal does not only depend on the number of seeds dispersed per scat but also on the abundance of the disperser^10^. All else being equal, local declines of brown bear populations due to harvesting and habitat loss can, therefore, be expected to reduce the quantity component of seed dispersal services provided by the species. Given that the effect of each individual brown bear is relatively large compared to the per individual effect of smaller-bodied seed dispersers (e.g., birds or small mammals), the potential effects of small population declines can translate into pronounced effects on seed dispersal processes and services. To our knowledge, there are no published studies about the relative contribution of brown bears to the total seed rain in comparison to other dispersers. However, preliminary results suggest that brown bears may disperse up to 90% of the total seeds mobilised by the whole frugivore community in European alpine forests (authors’ unpublished data). Asiatic black bears have been shown to be major seed dispersers in relation to other species in mixed temperate forests in Japan^47,48^. However, these are local studies and the relative importance of brown bears as seed dispersers may differ depending on the population densities of bears and other frugivores. In addition to the effects of local population declines, human activities can also compromise seed dispersal processes^49^. Anthropogenic food resources (i.e. any food resource derived from human activities) and artificial feeding (i.e. intentional food provisioning to wildlife by humans) may jeopardise the seed dispersal services provided by brown bears by affecting habitat selection, movement behaviour and dietary preferences^50–52^, as it has been already suggested for other carnivore species^53^.

The germination experiments showed that brown bear ingestion improves the ability of seeds to germinate when compared to seeds germinating from whole fruits and that it does not reduce seed germination in comparison to manually depulped fruits. These results are in line with previous works^54–56^ and suggest that the passage of fruits through the bears’ gut is beneficial for germination by removing the seeds from the pulp without harming the seeds^57^. Additionally, we first showed that bears rarely damaged the seeds of most fleshy fruits. Therefore, brown bears do not differ in the quality of their seed dispersal services from other major disperser guilds including birds, lizards, bats, mesocarnivores or elephants^45,54,58,59^. Thus, brown bears can be considered as legitimate seed dispersers also from a qualitative point of view. However, to fully understand the quality component of seed dispersal services provided by brown bears more information about the post-dispersal stages, such as the characteristics of the microhabitats in which brown bears defecate the seeds, is required. In this line, preliminary studies suggest that defecation of seeds in the surroundings of bears’ resting sites might be beneficial for plant recruitment, especially in the case of clonal species^56^. Seedling establishment is usually infrequent in these species and usually restricted to “windows of opportunity” (i.e. spatially or temporally unpredictable conditions in which seedling establishment is possible within stands of conspecific adults)^60^. Brown bears usually defecate next to their resting sites, where they dig and create local disturbances in the ground characterized by open top-soil with no or little vegetation^56^. These small disturbances might be essential for seedling recruitment of clonal plants as they expose the defecated seeds to the perfect conditions for germination, representing a valid example of these windows of opportunity. In addition, processes such as secondary seed dispersal and seed or seedling predation might be relevant if secondary dispersers or predators are attracted to bear scats containing large amounts of seeds^61,62^. Secondary dispersal may enhance germination probabilities by decreasing seed densities and, thus, seed competition, in spots with high densities of seeds such as bear scats. Additionally, secondary seed dispersers usually dig the soil and create small disturbances to relocate the seeds, creating the perfect environment for seedling establishment^62^. These aspects related to the quality of seed dispersal services provided by brown bears in comparison to other sympatric seed dispersers are relevant topics for future research.

The quality component of seed dispersal is not only related to fruit handling and gut treatment, but also to animal movement^10^. Brown bear daily displacement in the Carpathian Mountains, taken as the straight-line distance between two most distant locations during a 24 h period, is on average 2–4 km, but can reach up to 30 km^63^. Seeds dispersed across such distances might facilitate the colonization of new areas, and might enhance genetic diversity via reduction of adult-sibling competition and via release from high pathogen pressure near adult plants^64^. However, to our knowledge, there is only one study discussing the potential seed dispersal kernel provided by brown bears. Lalleroni et al.^42^ found that brown bears in the Pyrenees move on average between 0.85 and 1.34 km every 6 h, that corresponds to the median gut retention time for a berry-based diet^65^. Other bear species, such as the Asiatic black bear *Ursus thibetanus,* are known to move more than half of the seeds they consume over 500 m and to disperse seeds up to 22 km away from the source^66^. Based on the average daily displacement and gut retention times, brown bears would disperse seeds two-to-three times farther than other species considered long-distance dispersers, such as martens *Martes spp*. and the Japanese macaque *Macaca fuscata* (mean and maximum dispersal distances around 400–500 and 1,200 m, respectively)^67–70^. In temperate regions, red foxes may disperse seeds over similar average distances, but their maximum seed dispersal distances are still up to eight times smaller than the ones of bears (half of the seeds dispersed more than 1,000 m away from the source and up to 2846 m for foxes)^66,69^. The relationship between body size and dispersal distance has been proven in various taxa^4,71^. Brown bears may, thus, provide unique dispersal services by moving seeds over large distances, similar to recognised megafaunal long-distance seed dispersers inhabiting tropical areas such as African savanna elephants, which carried half of the seeds over 2.5 kilometers^72^.

We have shown that brown bears are legitimate seed dispersers across its range, providing high quantity and quality dispersal services. We have also shown that brown bears consume large amounts of seeds from many different fleshy-fruited plant species, that the large majority of these seeds remains intact after gut passage, and that gut passage does not reduce and even enhances the ability of seeds to germinate. Given its large body size the brown bear has the potential to contribute substantially to long-distance seed dispersal. Many large bodied frugivores, including megafauna, have been extirpated since the fifteenth century and populations of the remaining species show 25% average decline in abundance^18^. The loss of large-bodied frugivores frequently causes a reduction of seed dispersal distances, genetic diversity and effective population sizes of plants^6,16^. Studies about megafaunal frugivores have often focused on their role as the exclusive dispersers of large seeds (“megafaunal fruit syndrome”) and have been conducted mostly in tropical areas^73^. However, seed size is not expected to be a constraint of frugivore-mediated seed dispersal in Mediterranean, temperate and boreal regions, where fleshy fruits containing large seeds are missing. Consequently, we suggest that the uniqueness of megafaunal frugivores inhabiting these areas should be evaluated in terms of their significant contributions to the total seed rain and dispersal distances. We conclude that in Mediterranean, temperate and boreal biomes, where other megafauna species are unlikely to be effective seed dispersers, brown bears are an integral part of seed disperser communities. Our results suggest that brown bears are unique legitimate megafaunal seed dispersers that play an essential, though overlooked, role in plant regeneration processes and ecosystem functioning.

## Methods

### Literature review: species richness and quantitative importance of fleshy fruits in brown bear diet

We compiled published information about brown bear diet to analyse both the diversity and the quantitative importance of fleshy fruits in brown bear diet worldwide. We searched Google Scholar database for articles containing data on brown bear diet using the following keyword string: “(bear* or *ursus or *arctos) and (food* or habit* or forag* or diet* or faec* or scat* or stomach*)”. The search yielded 13,900 hits for the period 1900–2016 and we screened the first 1000 results. For those studies identified as relevant we also checked the reference lists for additional publications. If for a given population several studies had been published based on partly overlapping data, we only considered the latest study to avoid pseudo replicates in the database. We selected only studies covering the whole active period in brown bears and with a resolution of the food items good enough to distinguish fruits from other food items. In total, we selected for analyses 70 studies published between 1969 and 2016 that contained information about the diet of brown bears from 96 study areas covering the entire distribution of the species (Europe = 25, Asia = 30 and North America = 41 areas, Fig. 1, Appendix S6 and S7). In 69 out of these 70 publications (93 out of the total 96 study areas) bear scats were the only or the major source of information, whereas only one study used exclusively stomachs from killed animals. Thus, information was based on samples being already dispersed by brown bears or potentially dispersed in the case of killed animals. All the selected studies contained information from at least 15 brown bear diet samples. For 85 of the 96 areas fulfilling the above criteria, we extracted or calculated the relative frequency of occurrence of fleshy fruits as the number of occurrences of fleshy-fruited plant species divided by the total number of occurrences of all food items considered. Please note that it is different from the frequency of occurrence, where the number of occurrences is divided by the total number of samples. Whenever possible, we extracted the identity of the fleshy-fruited plants consumed at family, genus and species level. For each plant taxa, we also noted the number of times that it was recorded across study areas (e.g. 10 means that a taxon had been recorded in 10 different study areas). We extracted the latitude and longitude of the study areas and assigned each area to one of the following terrestrial biomes: (1) tundra, (2) boreal forests/taiga, (3) temperate coniferous forests, (4) temperate broadleaf and mixed forests, (5) montane grasslands and shrublands, (6) Mediterranean forest, woodlands and scrubs and (7) deserts and xeric shrublands^74^. To assess whether the relative frequency of occurrence was a good indicator of the amount of fruits and seeds consumed by brown bears per study area we also extracted from the same articles the relative volume of fleshy fruits, defined as the average percentage of volume that fleshy fruits represented out of the total volume of a scat; this information was available in 46 out of 96 study areas.

### Germination experiment: quality of the seeds dispersed by brown bears

We selected eleven fleshy-fruited plant species to investigate the effects of brown bear ingestion on the proportion and speed of germination. We chose species that are commonly eaten by brown bears in temperate and boreal forests, including our study area, the Carpathian Mountains, based on both literature review and previous field inspections, which also facilitated fruit collection to perform the germination experiments. The selected species represented a gradient of propagule size -from small to large seeds-, the number of seeds per fruit, and fruit type and size. We obtained data on the average seed weight from the Seed Information Database of the Kew Royal Botanic Gardens (http://www.kew.org/data/sid/).

For each species, we collected ripe fruits from at least 20 individual plants in the Bieszczady Mountains (SE Poland), located in the North-Eastern part of the Carpathian Mountains, during August–October 2008. We mixed the fruits of each species and divided them into three different groups according to the following treatments: (1) whole fruit treatment: 100 entire fruits of each species planted, i.e. seeds with the pulp, (2) depulped seed treatment: 500 seeds manually extracted, i.e. seeds without pulp, and (3) bear treatment: 500 seeds recovered from fresh bear scats.

For the bear treatment, we fed three captive brown bears in the Warsaw Zoological Garden with ripe fruits of each of the eleven selected species. Bear feeding was conducted three times in total, once per month from August to October, depending on the ripening time of each species. We recovered bear scats up to 24 h after feeding, kept them in plastic bags in a refrigerator for transport and processed them in the lab to extract the seeds. We mixed scats from each of the three trials to eliminate the potential effects of individual bears and washed them through a sieve (0.5 mm mesh size) with running water. Then, we sorted 500 intact seeds of each plant species for the bear treatment. We kept seeds for the depulped and bear treatment in a refrigerator at 6 °C until all bear-treatment seeds from the eleven species were ready.

We sowed all seeds at the same time, between 13 and 18th October 2008. The whole fruits (with the pulp) were planted immediately after returning from the field in seedbeds (one fruit per pot), thus, before the feeding trials with captive bears. For the depulped and bear treatment, we planted one seed in each pot of the seedbed. We sowed seeds and fruits in potting soil (peat soil in the case of *Vaccinium myrtillus*) in open-air seedbeds at the Krakow Botanical Garden, therefore, in outdoor conditions. The seeds stayed outside, being covered by snow during winter. The seedbeds were distributed together on concrete ground and covered with a mesh lid to prevent seed predation by rodents and birds. We checked the seedbeds from early April to late June in 2009 and 2010 at intervals of three to seven days, until no further germination was observed. We watered seeds and seedlings regularly depending on weather conditions. We conducted visits also during winter to control the state of the seedbeds. We noted the date and the germination in each pot and seedbed during each inspection (17 and 18 inspections in 2009 and 2010, respectively). In total, we monitored the germination of 12,100 seeds from early April 2009 to late June 2010.

Additionally, we collected brown bear scats in the Bieszczady Mountains in 2008–2010 as part of a larger project. We selected a subsample of 100 scats containing seeds of fleshy fruits to estimate the amount of seeds dispersed per scat. The scats were soaked in water with detergent, washed through a sieve, dried and weighed. Each scat was divided in five parts, two of them were randomly chosen, weighed and examined to count the total number of seeds and the number of damaged seeds (broken or crashed). We estimated the total number of seeds and the fraction of seeds damaged for the whole weight of the scat. Additionally, in each of the 11 selected fleshy-fruited plant species, we counted the number of seeds in 30 randomly chosen fruits.

### Data analyses

We used generalized linear models to analyse the effects of the biome on the relative frequency of occurrence and on the number of species and genera of fleshy fruits consumed by brown bears. We fitted the model of the relative frequency of occurrence to a quasibinomial distribution and logit-link function and both models of the number of species and genera to a Poisson distribution and log-link function. We excluded deserts from the analyses because only one study area was located in this biome (Fig. 1, Table 1).

We constructed a standardized study area × plant genus interaction matrix with the relative frequency of occurrence for each genus and included the study areas for which such data was available (66 out of 96 study areas). We performed a two-dimensional non-metric multidimensional scaling ordination to visualize differences and similarities in the contribution of the fleshy-fruited plant genera eaten by brown bears among the different biomes. We, then, performed a post-hoc permutational multivariate analysis of variance (permanova test) to check statistical differences in the contribution of the different fleshy-fruited plant genera among biomes.

We used data from the germination experiments to analyse the effects of bear ingestion on seed germination proportions and speed. Specifically, we calculated the mean germination times in 2009 and 2010 separately, and the proportion of seeds germinated at the end of the experiment for each combination of the eleven fleshy-fruited plant species and the three treatments. The mean germination time is often used as a proxy for the germination speed and it is measured as the weighted mean of the germination time (mean germination time = Σ(n × D)/Σn, where n is the number of seeds germinated on day D of the experiment)^75^. Based on evidence from the experiment, we considered April 1st and June 30th as the start and the end of the germination period in each year.

We performed generalized linear mixed effects models to test the effects of germination treatments on the proportion of seeds germinated at the end of the experiment (2010) and on the mean germination time during the first year (2009). We excluded *Viburnum opulus* and *Crataegus monogyna* from the analysis of mean germination time because the first species did not germinate during 2009 and the second germinated very little (Appendix S4 and S5); just some of the seeds germinated before the first inspection in 2010. We fitted the proportion of seeds germinated to a binomial distribution and logit-link function and the mean germination time to a Poisson distribution and log-link function. We included treatment (bear, depulped and whole fruit) as a fixed factor and plant species as a random intercept. We used Spearman correlation to analyse the relation between the weight of the seeds of each species and the proportion of intact seeds after brown bear ingestion.

We used the R statistical environment (version 3.4.0, R Development Core Team 2017) to perform all the statistical analyses. We used the R packages *lme4*^76^ for the implementation of the generalized linear mixed effects models, *Vegan*^77^ for the nonmetric multidimensional scaling ordination and the permanova test and *SeedCalc*^78^ to calculate mean germination times.

## Supplementary information

Supplementary Information.

## Data Availability

The datasets generated and analysed during the current study are available from the corresponding author upon reasonable request.
