# Supplementary material for: The role of the brown bear Ursus arctos as a legitimate megafaunal seed disperser
Source: Sci Rep. 2021 Jan 14;11:1282. doi: 10.1038/s41598-020-80440-9 (PMC7809135; doi:10.1038/s41598-020-80440-9)
Supplement: Supplementary file 1 — Supplementary Information. [file 41598_2020_80440_MOESM1_ESM.docx]

Supplementary material for:

The role of the brown bear *Ursus arctos* as a legitimate megafaunal seed disperser

Scientific Reports

Alberto García-Rodríguez, Jörg Albrecht, Sylwia Szczutkowska, Alfredo Valido, Nina Farwig, Nuria Selva

This file includes:

**Appendix S1.** List of fleshy-fruited plant taxa (families, genera and species) consumed by brown bears and number of study areas (n= 96) where each taxon was recorded as brown bear food.

**Appendix S2.** Summary statistics (Estimate – Est., Standard Error – S.E., t value – t, and p-value) for the three generalized linear models performed to test the effects of the biome on the number of taxa (genera and species) and the relative frequency of occurrence of fleshy fruits consumed by brown bears. The intercept corresponds to Montane grasslands & shrublands. Deserts are excluded due to small sample size (n = 1 study area). The list of study areas and their corresponding biomes are provided in Appendix S1.

**Appendix S3.** Summary statistics (Estimate – Est., Standard Error – S.E., z value – z, and p-value) for the two generalized linear mixed models performed to test the effects of the germination treatment (seeds ingested by bears and recovered from the scats, manually depulped seeds, and seeds embedded within the whole fruit) on the proportion of seeds germinated at the end of the 2-year experiment and on the mean germination time during the first year of germination of eleven fleshy-fruited plant species commonly eaten by brown bears in Eurasian temperate regions. Fleshy-fruited plant species, final percentages of seeds germinated and mean germination times are given in Table 2. *Significant values are presented in bold.

**Appendix S4.** Percentage of seeds germinated at the end of the 2-year experiment and mean germination times (number of days elapsed since April 1^st^) during the first year of germination for eleven fleshy-fruited plant species eaten by brown bears in Eurasian temperate regions and under three different germination treatments (seeds ingested by bears and recovered from the scats, manually depulped seeds, and seeds embedded within the whole fruit). The percentage of seeds germinated in 2010 is based on those seeds that did not germinate in 2009. NA values are present for those combinations of species and treatments for which mean germination times were not possible to calculate.

**Appendix S5.** Germination curves for eleven fleshy-fruited plant species eaten by brown bears in temperate regions in Eurasia and under three germination treatments: (1) seeds ingested by bears and recovered from the scats (empty dots), (2) manually depulped seeds (triangles) and (3) seeds embedded within the whole fruit (crosses) during 2009 (left pannel)- and 2010 (right pannel). Germination inspections started on April 1^st^ and finished on June 30^th^ each year.

**Appendix S6**. ID, country, biome, latitude and longitude (in degrees) of the study areas (n = 96) from which data on brown bears diet were gathered. Number of species and genera, relative frequency of occurrence (rF) and relative volume (rV) of fleshy fruits in brown bear diet, number of samples analyzed and the reference from which data were collected are also provided for each study area.

**Appendix S7.** References (n=70) from which data on brown bear diet across its distribution range, including 96 study areas, were gathered. The ID of the study areas of each publication is also provided.

**Appendix S1**. List of fleshy-fruited plant taxa (families, genera and species) consumed by brown bears and number of study areas (n= 96) where each taxon was recorded as brown bear food.

| **Family** | **Study areas** | **Genus** | **Study areas** | **Species** | **Study areas** |
| --- | --- | --- | --- | --- | --- |
| Rosaceae | 62 | Rubus | 43 | *Empetrum nigrum* | 20 |
| Ericaceae | 47 | Vaccinium | 40 | *Vaccinium myrtillus* | 19 |
| Cornaceae | 15 | Prunus | 31 | *Vaccinium vitis-idaea* | 17 |
| Grossulariaceae | 15 | Empetrum | 23 | *Rubus idaeus* | 16 |
| Vitaceae | 13 | Malus | 23 | *Vaccinium uliginosum* | 13 |
| Rhamnaceae | 11 | Sorbus | 22 | *Rubus chamaemorus* | 11 |
| Actinidiaceae | 10 | Rosa | 19 | *Actinidia arguta* | 10 |
| Adoxaceae | 10 | Cornus | 15 | *Cornus mas* | 9 |
| Araliaceae | 10 | Ribes | 15 | *Sorbus aucuparia* | 9 |
| Elaeagnaceae | 9 | Arctostaphylos | 14 | *Shepherdia canadensis* | 8 |
| Moraceae | 5 | Pyrus | 14 | *Vitis coignetiae* | 8 |
| Berberidaceae | 4 | Vitis | 13 | *Prunus avium* | 7 |
| Caprifoliaceae | 4 | Actinidia | 10 | *Ribes triste* | 7 |
| Cupressaceae | 3 | Aralia | 9 | *Rosa canina* | 7 |
| Cucurbitaceae | 2 | Rhamnus | 9 | *Aralia cordata* | 6 |
| Aquifoliaceae | 1 | Shepherdia | 9 | *Prunus domestica* | 6 |
| Ephedraceae | 1 | Viburnum | 9 | *Prunus spinosa* | 6 |
| Liliaceae | 1 | Crataegus | 8 | *Prunus ssiori* | 6 |
| Nitrariaceae | 1 | Fragaria | 7 | *Actinidia kolomikta* | 5 |
| Oleaceae | 1 | Amelanchier | 5 | *Arctostaphylos rubra* | 5 |
| Santalaceae | 1 | Berberis | 4 | *Arctostaphylos uva-ursi* | 5 |
| Solanaceae | 1 | Lonicera | 4 | *Malus sylvestris* | 5 |
| Taxaceae | 1 | Ficus | 3 | *Pyrus communis* | 4 |
| Thymelaeaceae | 1 | Juniperus | 3 | *Rhamnus alpina* | 4 |
|  |  | Morus | 3 | *Ribes dikuscha* | 4 |
|  |  | Arbutus | 2 | *Vaccinium oxycoccos* | 4 |
|  |  | Frangula | 2 | *Vitis vinifera* | 4 |
|  |  | Sambucus | 2 | *Actinidia polygama* | 3 |
|  |  | Citrullus | 1 | *Amelanchier ovalis* | 3 |
|  |  | Daphne | 1 | *Cornus sericea* | 3 |
|  |  | Ephedra | 1 | *Crataegus monogyna* | 3 |
|  |  | Ilex | 1 | *Ficus carica* | 3 |
|  |  | Kalopanax | 1 | *Fragaria vesca* | 3 |
|  |  | Nitraria | 1 | *Prunus cerasifera* | 3 |
|  |  | Olea | 1 | *Sorbus aria* | 3 |
|  |  | Oplopanax | 1 | *Vaccinium scoparium* | 3 |
|  |  | Oxycoccus | 1 | *Viburnum opulus* | 3 |
|  |  | Solanum | 1 | *Amelanchier alnifolia* | 2 |
|  |  | Streptopus | 1 | *Arbutus unedo* | 2 |
|  |  | Taxus | 1 | *Arctostaphylos alpinus* | 2 |
|  |  | Ventia | 1 | *Berberis aristata* | 2 |
|  |  | Viscum | 1 | *Frangula alnus* | 2 |
|  |  |  |  | *Malus domestica* | 2 |
|  |  |  |  | *Malus pumila* | 2 |
|  |  |  |  | *Morus australis* | 2 |
|  |  |  |  | *Prunus cornuta* | 2 |
|  |  |  |  | *Prunus mahaleb* | 2 |
|  |  |  |  | *Prunus padus* | 2 |
|  |  |  |  | *Prunus persica* | 2 |
|  |  |  |  | *Prunus salicina* | 2 |
|  |  |  |  | *Prunus virginiana* | 2 |
|  |  |  |  | *Rosa acicularis* | 2 |
|  |  |  |  | *Rubus fruticosus* | 2 |
|  |  |  |  | *Rubus sachalinensis* | 2 |
|  |  |  |  | *Sambucus racemosa* | 2 |
|  |  |  |  | *Sorbus commixta* | 2 |
|  |  |  |  | *Sorbus domestica* | 2 |
|  |  |  |  | *Vaccinium globulare* | 2 |
|  |  |  |  | *Viburnum cotinifolium* | 2 |
|  |  |  |  | *Viburnum furcatum* | 2 |
|  |  |  |  | *Aralia elata* | 1 |
|  |  |  |  | *Berberis repens* | 1 |
|  |  |  |  | *Citrullus lanatus* | 1 |
|  |  |  |  | *Cornus controversa* | 1 |
|  |  |  |  | *Crataegus douglasii* | 1 |
|  |  |  |  | *Fragaria virginiana* | 1 |
|  |  |  |  | *Ilex aquifolium* | 1 |
|  |  |  |  | *Juniperus communis* | 1 |
|  |  |  |  | *Juniperus horizontalis* | 1 |
|  |  |  |  | *Kalopanax septemlobus* | 1 |
|  |  |  |  | *Lonicera involucrata* | 1 |
|  |  |  |  | *Lonicera xylosteum* | 1 |
|  |  |  |  | *Morus alba* | 1 |
|  |  |  |  | *Olea europaea* | 1 |
|  |  |  |  | *Oplopanax horridus* | 1 |
|  |  |  |  | *Oxycoccus microcarpus* | 1 |
|  |  |  |  | *Prunus cerasus* | 1 |
|  |  |  |  | *Prunus cocomilia* | 1 |
|  |  |  |  | *Prunus sargentii* | 1 |
|  |  |  |  | *Rhamnus alnifolia* | 1 |
|  |  |  |  | *Rhamnus cathartica* | 1 |
|  |  |  |  | *Rhamnus virgata* | 1 |
|  |  |  |  | *Ribes bracteosum* | 1 |
|  |  |  |  | *Ribes montigenum* | 1 |
|  |  |  |  | *Ribes oxyacanthoides* | 1 |
|  |  |  |  | *Ribes uva-crispa* | 1 |
|  |  |  |  | *Rosa majalis* | 1 |
|  |  |  |  | *Rubus arcticus* | 1 |
|  |  |  |  | *Rubus caesius* | 1 |
|  |  |  |  | *Rubus spectabilis* | 1 |
|  |  |  |  | *Rubus ulmifolius* | 1 |
|  |  |  |  | *Shepherdia argentea* | 1 |
|  |  |  |  | *Solanum nigrum* | 1 |
|  |  |  |  | *Sorbus matsumurana* | 1 |
|  |  |  |  | *Sorbus sambucifolia* | 1 |
|  |  |  |  | *Streptopus lanceolatus* | 1 |
|  |  |  |  | *Taxus cuspidata* | 1 |
|  |  |  |  | *Vaccinium caespitosum* | 1 |
|  |  |  |  | *Vaccinium membranaceum* | 1 |
|  |  |  |  | *Viburnum edule* | 1 |
|  |  |  |  | *Viscum album* | 1 |

**Appendix S2.** Summary statistics (Estimate – Est., Standard Error – S.E., z value – z, t value – t, and p-value) for the three generalized linear models performed to test the effects of the biome on the number of taxa (genera and species) and the relative frequency of occurrence of fleshy fruits consumed by brown bears. The intercept corresponds to Montane grasslands & shrublands. Deserts are excluded due to small sample size (n = 1 study area). The list of study areas and their corresponding biomes are provided in Appendix S1.

|  | **Number of genera** | | | | **Number of species** | | | | **Relative frequency of occurrence** | | | |
| --- | --- | --- | --- | --- | --- | --- | --- | --- | --- | --- | --- | --- |
| **Biome** | **Est.** | **S.E.** | **z** | **p-value** | **Est.** | **S.E.** | **z** | **p-value** | **Est.** | **S.E.** | **t** | **p-value** |
| Intercept | 1.61 | 0.31 | 5.09 | <0.001 | 1.61 | 0.31 | 5.09 | <0.001 | -1.15 | 0.47 | -2.44 | 0.017 |
| Tundra | -0.51 | 0.35 | -1.45 | 0.147 | -0.38 | 0.35 | -1.08 | 0.278 | 0.08 | 0.51 | 0.15 | 0.877 |
| Boreal forests & taiga | -0.38 | 0.34 | -1.11 | 0.265 | -0.03 | 0.34 | -0.08 | 0.936 | 0.16 | 0.50 | 0.33 | 0.743 |
| Temperate coniferous forests | -0.21 | 0.33 | -0.65 | 0.514 | -0.13 | 0.33 | -0.39 | 0.697 | -0.48 | 0.49 | -0.97 | 0.335 |
| Temperate mixed & broadleaf forests | 0.04 | 0.33 | 0.12 | 0.904 | 0.10 | 0.32 | 0.31 | 0.756 | 0.09 | 0.49 | 0.20 | 0.841 |
| Mediterranean forests, woodlands & scrubs | 0.34 | 0.37 | 0.91 | 0.361 | 0.37 | 0.37 | 1.01 | 0.311 | 0.94 | 0.55 | 1.71 | 0.092 |

**Appendix S3.** Summary statistics (Estimate – Est., Standard Error – S.E., z value – z, and p-value) for the two generalized linear mixed models performed to test the effects of the germination treatment (seeds ingested by bears and recovered from the scats, manually depulped seeds, and seeds embedded within the whole fruit) on the proportion of seeds germinated at the end of the 2-year experiment and on the mean germination time during the first year of germination of eleven fleshy-fruited plant species commonly eaten by brown bears in Eurasian temperate regions. Fleshy-fruited plant species, final percentages of seeds germinated and mean germination times are given in Table 2. *Significant values are presented in bold.

|  | **Proportion of seeds germinated** | | | | **Mean germination time** | | | |
| --- | --- | --- | --- | --- | --- | --- | --- | --- |
|  | **Est.** | **S.E.** | **z** | **p-value** | **Est.** | **S.E.** | **z** | **p-value** |
| **Fixed factors** |  |  |  |  |  |  |  |  |
| Intercept | -2.30 | 1.05 | -2.19 | **0.028*** | 3.03 | 0.19 | 16.10 | **<0.001*** |
| Bear | 2.86 | 1.22 | 2.34 | **0.019*** | -0.01 | 0.10 | -0.03 | 0.9 |
| Depulped | 2.48 | 1.21 | 2.05 | **0.040*** | 0.05 | 0.10 | 0.54 | 0.591 |
| **Random factor** |  |  |  |  |  |  |  |  |
| Species | 0.00 | 0.00 |  |  | 0.26 | 0.51 |  |  |

**Appendix S4**. Percentage of seeds germinated at the end of the 2-year experiment and mean germination times (number of days elapsed since April 1^st^) during the first year of germination for eleven fleshy-fruited plant species eaten by brown bears in Eurasian temperate regions and under three different germination treatments (seeds ingested by bears and recovered from the scats, manually depulped seeds, and seeds embedded within the whole fruit). The percentage of seeds germinated in 2010 is based on those seeds that did not germinate in 2009. NA values are present for those combinations of species and treatments for which mean germination times were not possible to calculate.

|  |  |  | **2009** | | **2010** | |
| --- | --- | --- | --- | --- | --- | --- |
| **Species** | **No. seeds per fruit** | **Treatment** | **Percentage germinated (%)** | **Mean germination time (days)** | **Percentage germinated (%)** | **Mean germination time (days)** |
| ***Rosa sp.*** | 25 | Whole | 0.40 | 20.30 | 7.27 | 20.93 |
|  |  | Depulped | 4.80 | 25.04 | 50.00 | 17.93 |
|  |  | Bear | 2.20 | 21.45 | 53.37 | 19.05 |
| ***Frangula alnus*** | 2.5 | Whole | 37.60 | 29.22 | 0 | NA |
|  |  | Depulped | 61.20 | 26.26 | 0 | NA |
|  |  | Bear | 71.80 | 26.95 | 0 | NA |
| ***Vaccinium myrtillus*** | 52 | Whole | 0.46 | 49.17 | 0 | NA |
|  |  | Depulped | 30.00 | 49.51 | 0 | NA |
|  |  | Bear | 22.40 | 49.23 | 0 | NA |
| ***Rubus fruticosa*** | 29 | Whole | 7.83 | 39.42 | 13.40 | 19.42 |
|  |  | Depulped | 18.40 | 58.21 | 33.33 | 19.29 |
|  |  | Bear | 41.00 | 49.88 | 32.41 | 8.99 |
| ***Sambucus nigra*** | 3 | Whole | 35.67 | 12.99 | 1.03 | 1.38 |
|  |  | Depulped | 61.20 | 22.07 | 10.20 | 2.51 |
|  |  | Bear | 74.80 | 15.21 | 11.90 | 2.04 |
| ***Sorbus aucuparia*** | 2.2 | Whole | 0.91 | NA | 46.33 | 19.56 |
|  |  | Depulped | 43.80 | 14.22 | 3.91 | 2.23 |
|  |  | Bear | 64.60 | 12.10 | 14.69 | 2.76 |
| ***Prunus spinosa*** | 1 | Whole | 2.00 | 27.50 | 44.90 | 19.15 |
|  |  | Depulped | 62.60 | 15.70 | 35.82 | 4.87 |
|  |  | Bear | 68.00 | 19.81 | 36.87 | 4.18 |
| ***Prunus avium*** | 1 | Whole | 33.00 | 8.67 | 0 | NA |
|  |  | Depulped | 27.20 | 14.01 | 0 | NA |
|  |  | Bear | 16.20 | 14.86 | 5.25 | 4.59 |
| ***Malus sylvestris*** | 4.2 | Whole | 11.43 | 16.40 | 2.69 | 12.12 |
|  |  | Depulped | 69.80 | 10.61 | NA | NA |
|  |  | Bear | 88.20 | 9.75 | 5.01 | 1.11 |
| ***Viburnum opulus*** | 1 | Whole | 0 | NA | 65.00 | NA |
|  |  | Depulped | 0 | NA | 55.40 | NA |
|  |  | Bear | 0 | NA | 49.60 | NA |
| ***Crataegus monogyna*** | 1 | Whole | 0 | NA | 20.00 | 12.90 |
|  |  | Depulped | 2.00 | NA | 24.28 | 15.73 |
|  |  | Bear | 0 | NA | 24.80 | 15.40 |

**Appendix S5**. Germination curves for eleven fleshy-fruited plant species eaten by brown bears in temperate regions in Eurasia and under three germination treatments: (1) seeds ingested by bears and recovered from the scats (empty dots), (2) manually depulped seeds (triangles) and (3) seeds embedded within the whole fruit (crosses) during 2009 (left pannel)- and 2010 (right pannel). Germination inspections started on April 1^st^ and finished on June 30^th^ each year.


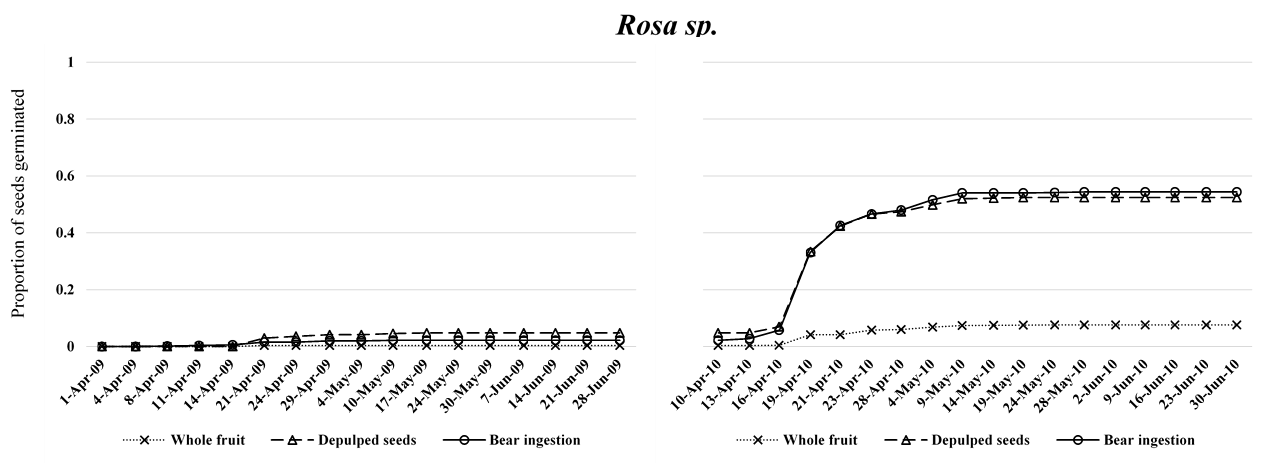


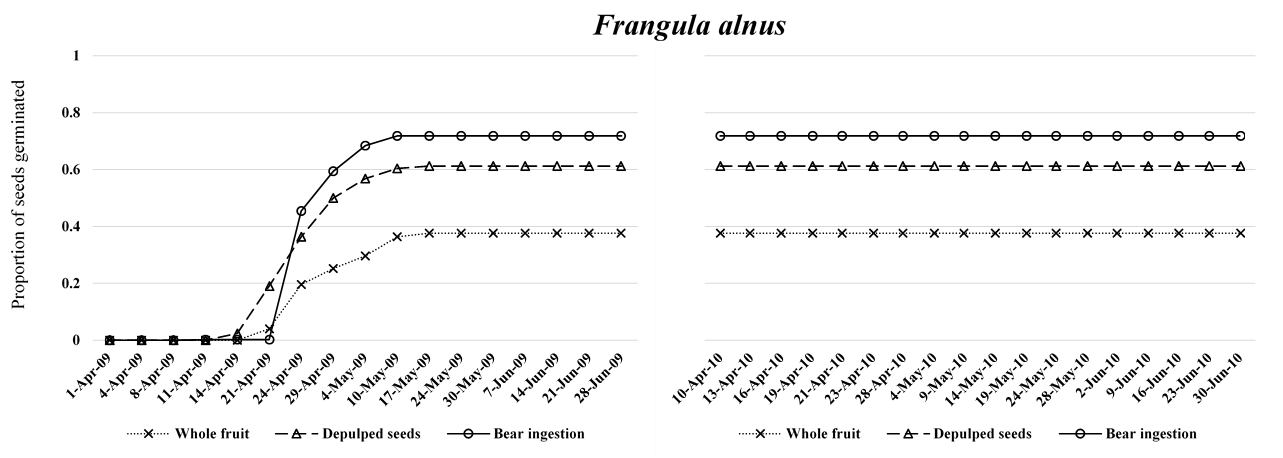


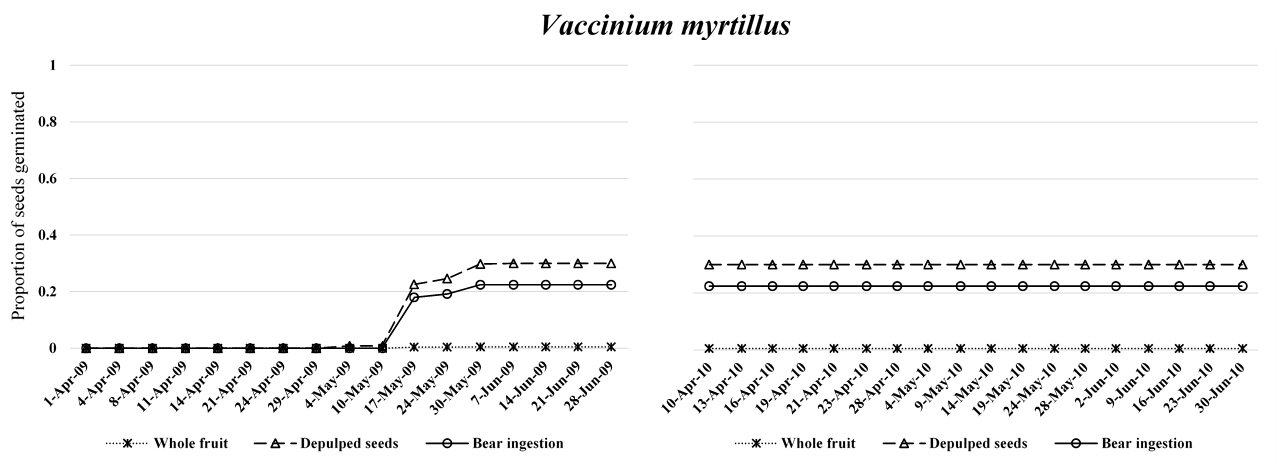


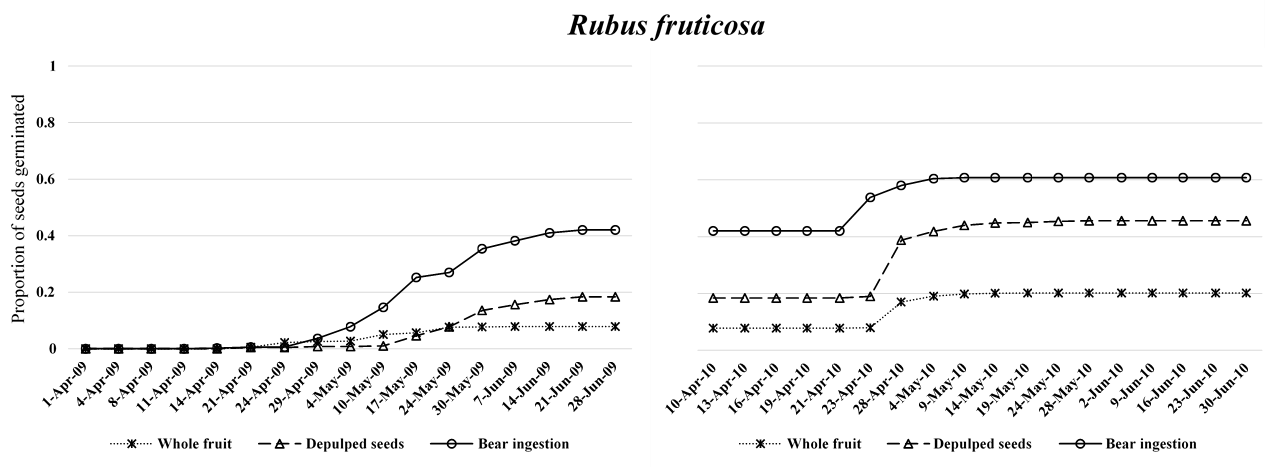


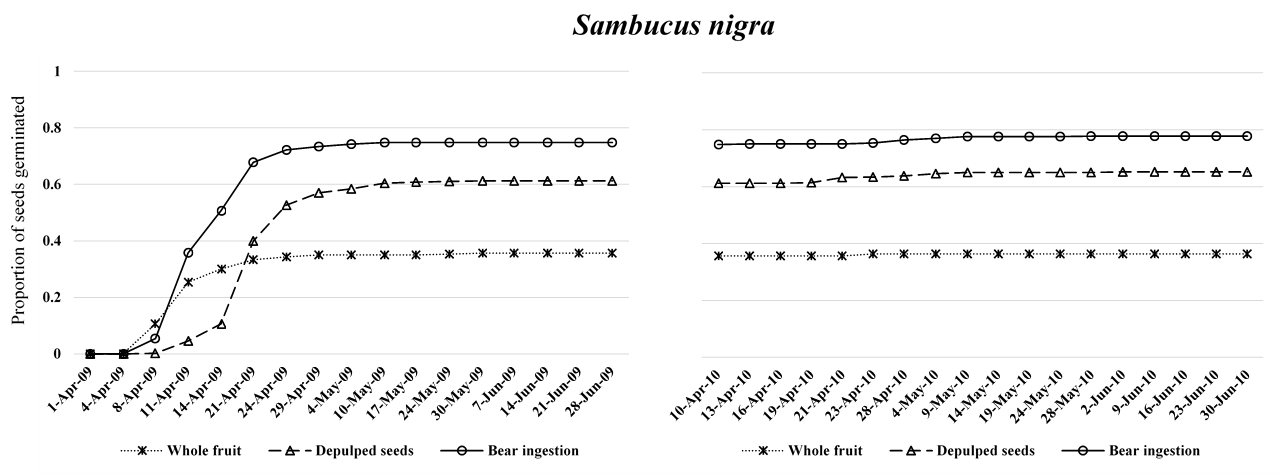


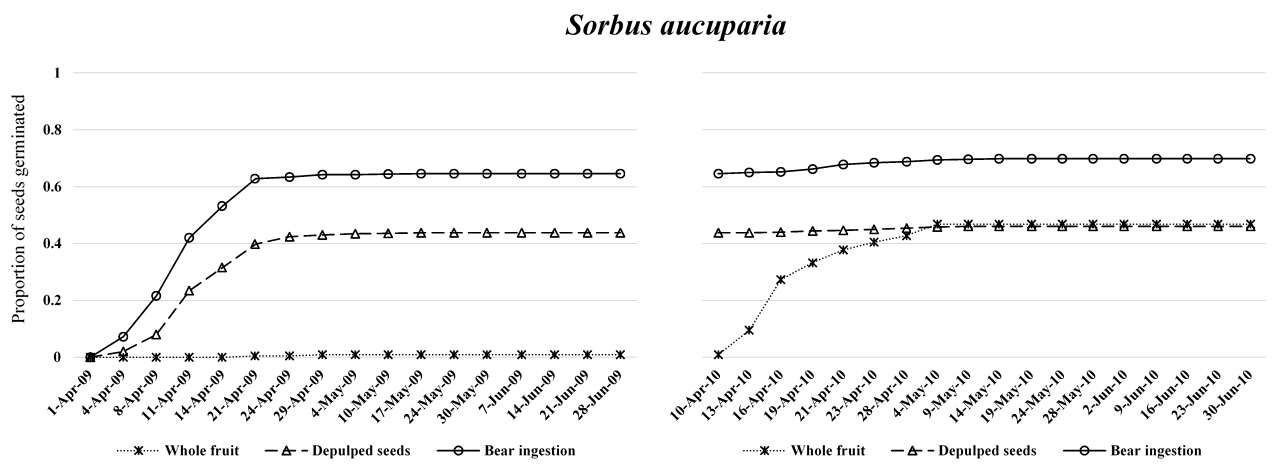


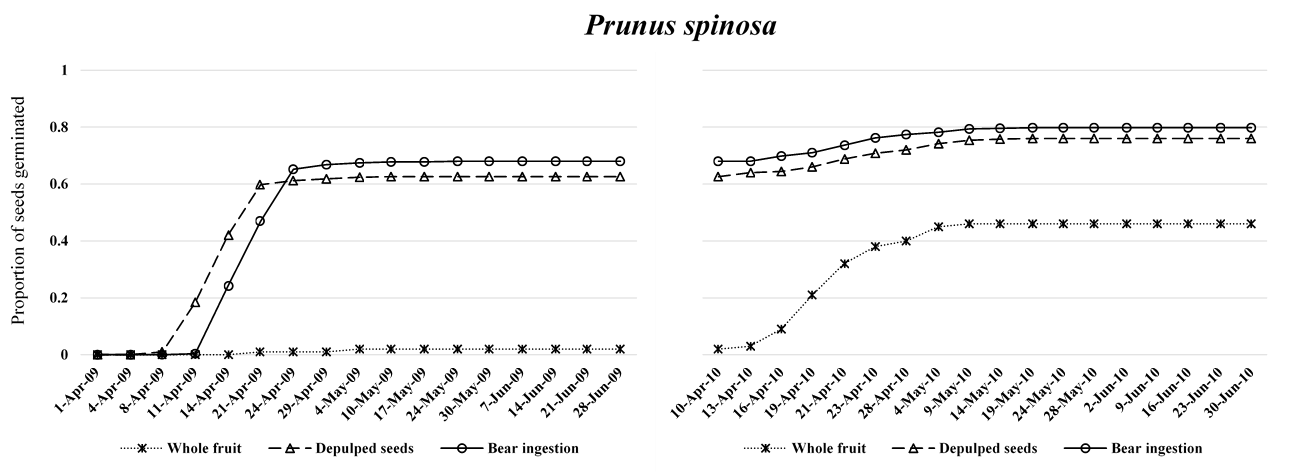


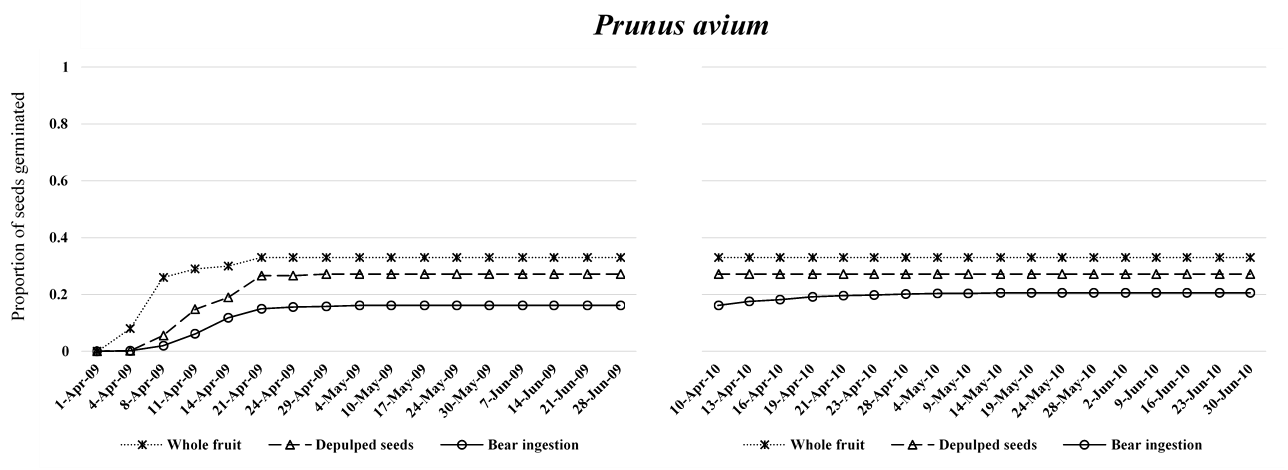


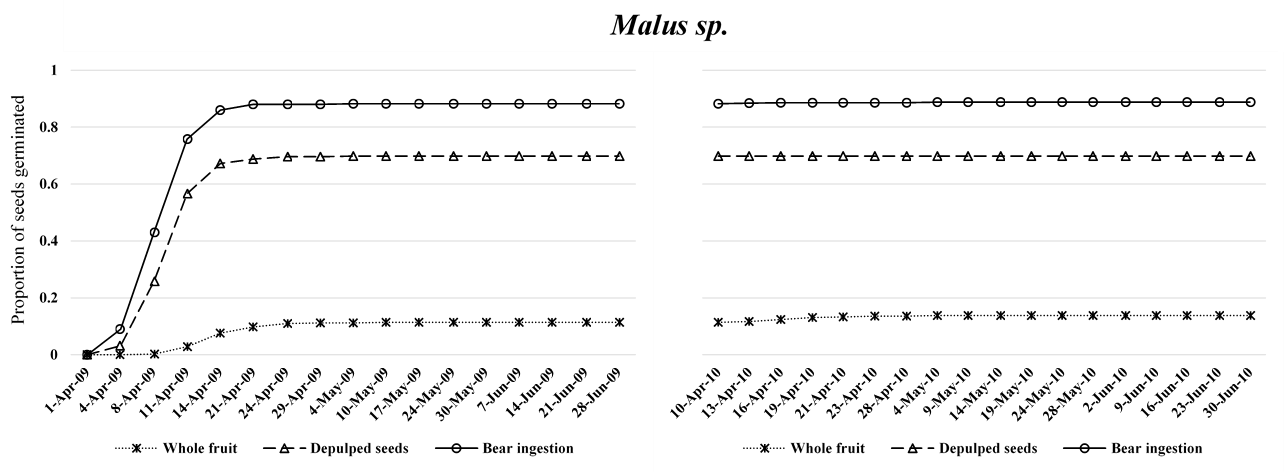


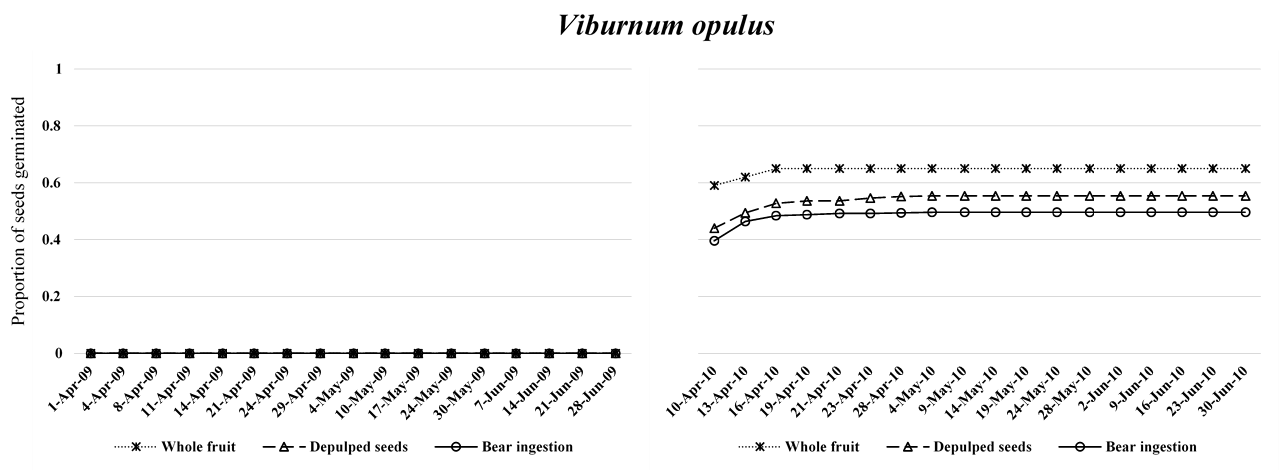


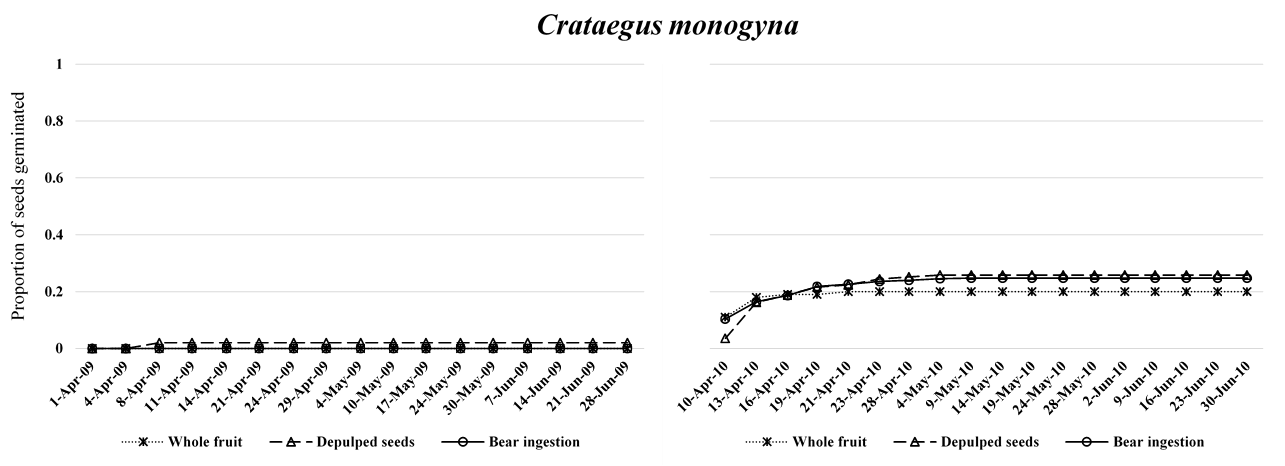


**Appendix S6.** ID, country, biome, latitude and longitude (in degrees) of the study areas (n = 96) from which data on brown bears diet were gathered. Number of species and genera, relative frequency of occurrence (rF) and relative volume (rV) of fleshy fruits in brown bear diet, number of samples analyzed and the reference from which data were collected are also provided for each study area.

| ID | **Country** | **Biome** | **Latitude** | **Longitude** | **Species** | **Genera** | **rF** | **rV** | **No. samples** | **Reference** |
| --- | --- | --- | --- | --- | --- | --- | --- | --- | --- | --- |
| 1 | Russia | Tundra | 68.0 | -178.3 | 4 | 3 | 0.27 | NA | 102 | Chernjavskij & Krechmar (1993) |
| 2 | Russia | Tundra | 67.9 | -177.9 | 4 | 3 | 0.27 | NA | 102 | Krechmar (1995) |
| 3 | Russia | Tundra | 64.9 | -174.8 | 3 | 2 | 0.14 | NA | 55 | Chernjavskij & Krechmar (1993) |
| 4 | Russia | Tundra | 65.0 | -173.4 | 3 | 2 | 0.14 | NA | 55 | Krechmar (1995) |
| 5 | USA | Tundra | 69.3 | -153.5 | 4 | 1 | NA | NA | NA | Reynolds (1980) |
| 6 | USA | Tundra | 63.3 | -151.1 | 4 | 2 | 0.37 | NA | 810 | Murie (1981) |
| 7 | USA | Tundra | 63.5 | -150.0 | 3 | 2 | 0.28 | NA | 196 | Stelmock & Dean (1986) |
| 8 | USA | Tundra | 63.6 | -149.7 | 1 | 1 | 0.53 | NA | 406 | Stelmock & Dean (1986) |
| 9 | USA | Tundra | 69.1 | -144.0 | 1 | 1 | 0.16 | NA | 113 | Phillips (1987) |
| 10 | Canada | Tundra | 69.2 | -140.1 | 5 | 3 | 0.19 | 0.17 | 321 | MacHutchon & Wellwood (2003) |
| 11 | Canada | Boreal forests & taiga | 61.0 | -138.0 | 2 | 2 | 0.27 | NA | 128 | Pearson (1975) |
| 12 | Canada | Temperate coniferous forests | 53.0 | -127.0 | 10 | 8 | NA | NA | NA | Hamilton & Bunnell (1987) |
| 13 | Canada | Temperate coniferous forests | 53.3 | -118.5 | 8 | 6 | NA | NA | 331 | Cristescu et al. (2015) |
| 14 | Canada | Temperate coniferous forests | 53.0 | -117.0 | 1 | 1 | NA | NA | 665 | Munro et al. (2006) |
| 15 | Canada | Temperate coniferous forests | 51.2 | -115.6 | 3 | 2 | 0.23 | NA | 381 | Hamer & Herrero (1987) |
| 16 | USA | Temperate coniferous forests | 49.3 | -114.9 | 1 | 1 | 0.23 | 0.01 | 306 | Mace & Jonkel (1986) |
| 17 | Canada | Temperate coniferous forests | 49.0 | -114.9 | 1 | 1 | 0.23 | 0.33 | 1100 | McLellan & Hovey (1995) |
| 18 | USA | Temperate coniferous forests | 47.0 | -114.0 | 16 | 9 | 0.13 | 0.19 | 1094 | Aune & Kasworm (1989) |
| 19 | USA | Temperate coniferous forests | 47.0 | -114.0 | 7 | 4 | 0.15 | 0.29 | 144 | Aune (1985) |
| 20 | USA | Temperate coniferous forests | 47.0 | -114.0 | 1 | 1 | 0.15 | 0.21 | 1094 | Aune (1994) |
| 21 | USA | Temperate coniferous forests | 47.4 | -113.9 | 1 | 1 | 0.18 | NA | 293 | Mace & Jonkel (1986) |
| 22 | USA | Temperate coniferous forests | 47.4 | -113.9 | 1 | 1 | NA | 0.23 | 177 | Servheen (1983) |
| 23 | USA | Temperate coniferous forests | 47.4 | -113.2 | 1 | 1 | 0.18 | NA | 140 | Mace & Jonkel (1986) |
| 24 | USA | Temperate coniferous forests | 47.8 | -112.7 | 1 | 1 | 0.19 | 0.01 | 417 | Mace & Jonkel (1986) |
| 25 | USA | Temperate coniferous forests | 44.6 | -110.6 | 2 | 2 | 0.01 | 0.14 | 3028 | Knight et al. (1982) |
| 26 | USA | Temperate coniferous forests | 44.6 | -110.6 | 1 | 1 | NA | 0.03 | 3938 | Mattson et al. (1991) |
| 27 | USA | Temperate coniferous forests | 44.6 | -110.5 | 2 | 2 | 0.1 | 0.17 | 487 | Craighead et al. (1995) |
| 28 | USA | Temperate coniferous forests | 44.6 | -110.5 | 3 | 3 | 0.03 | 0.03 | 615 | Mealey (1980) |
| 29 | Canada | Tundra | 64.5 | -110.5 | 5 | 2 | 0.38 | 0.14 | 169 | Gau et al. (2002) |
| 30 | Spain | Temperate broadleaf & mixed forests | 43.3 | -5.3 | 8 | 4 | 0.14 | 0.1 | 929 | Clevenger et al. (1992) |
| 31 | Spain | Temperate broadleaf & mixed forests | 43.3 | -5.3 | 11 | 4 | 0.27 | 0.28 | 1500 | Naves et al. (2006) |
| 32 | Spain | Temperate broadleaf & mixed forests | 43.0 | -5.0 | 10 | 3 | 0.19 | NA | 261 | Braña et al. (1987) |
| 33 | France | Temperate broadleaf & mixed forests | 43.0 | -0.5 | 4 | 3 | NA | 0.4 | 252 | Berducou et al. (1983) |
| 34 | France | Temperate broadleaf & mixed forests | 42.7 | 0.6 | 6 | 4 | 0.07 | NA | 89 | Lagalisse (2002) |
| 35 | Norway | Boreal forests & taiga | 61.0 | 10.0 | 3 | 2 | 0.25 | 0.37 | 134 | Elgmork & Kaasa (1992) |
| 36 | Italy | Temperate broadleaf & mixed forests | 41.8 | 13.8 | 15 | 7 | 0.3 | 0.38 | 2359 | Ciucci et al. (2014) |
| 37 | Italy | Temperate broadleaf & mixed forests | 41.8 | 13.8 | 11 | 6 | 0.35 | 0.47 | 328 | Di Domenico et al. (2012) |
| 38 | Italy | Temperate broadleaf & mixed forests | 41.8 | 13.9 | 1 | 1 | 0.34 | NA | 232 | Zunino & Herrero (1972) |
| 39 | Norway | Boreal forests & taiga | 64.0 | 14.0 | 3 | 2 | 0.13 | 0.13 | 118 | Dahle et al. (1998) |
| 40 | Sweden | Boreal forests & taiga | 64.0 | 14.0 | 2 | 1 | 0.23 | 0.36 | 148 | Dahle et al. (1998) |
| 41 | Slovenia | Temperate broadleaf & mixed forests | 45.9 | 14.4 | 10 | 3 | 0.17 | NA | 363 | Kavcic et al. (2015) |
| 42 | Slovenia | Temperate broadleaf & mixed forests | 46.1 | 14.5 | 1 | 1 | NA | 0.13 | 200 | Große et al. (2003) |
| 43 | Sweden | Boreal forests & taiga | 61.0 | 15.0 | 3 | 1 | 0.32 | 0.53 | 539 | Stenset et al. (2016) |
| 44 | Croatia | Temperate broadleaf & mixed forests | 44.9 | 15.6 | 10 | 4 | 0.31 | 0.33 | 95 | Cicnjak et al. (1987) |
| 45 | Slovakia | Temperate coniferous forests | 48.6 | 19.5 | 7 | 3 | 0.19 | 0.14 | 243 | Skuban et al. (2016) |
| 46 | Slovakia | Temperate coniferous forests | 49.2 | 19.9 | 8 | 3 | 0.28 | 0.28 | 373 | Rigg & Gorman (2005) |
| 47 | Poland | Temperate coniferous forests | 49.2 | 20.1 | 6 | 3 | 0.31 | NA | 68 | Jamnicky (1988) |
| 48 | Greece | Mediterranean forests, woodlands & scrubs | 40.9 | 21.0 | 5 | 2 | 0.53 | 0.53 | 223 | Mertzanis et al. (2000) |
| 49 | Poland | Temperate coniferous forests | 49.2 | 21.0 | 2 | 1 | 0.08 | 0.08 | 46 | Frackowiak & Gula (1992) |
| 50 | Greece | Mediterranean forests, woodlands & scrubs | 39.9 | 21.1 | 5 | 3 | 0.54 | NA | 343 | Mertzanis (1992) |
| 51 | Greece | Mediterranean forests, woodlands & scrubs | 39.9 | 21.1 | 12 | 6 | 0.14 | NA | 360 | Paralikidis et al. (2010) |
| 52 | Greece | Mediterranean forests, woodlands & scrubs | 39.9 | 21.6 | 7 | 3 | 0.58 | NA | 208 | Vlachos et al. (2000) |
| 53 | Slovakia | Temperate broadleaf & mixed forests | 48.0 | 22.0 | 4 | 1 | 0.34 | NA | 215 | Stofik et al. (2013) |
| 54 | Poland | Temperate coniferous forests | 49.3 | 22.5 | 5 | 2 | 0.23 | 0.29 | 184 | Frackowiak (1997) |
| 55 | Ukraine | Temperate broadleaf & mixed forests | 48.3 | 23.2 | 1 | 1 | 0.3 | NA | 401 | Vaisfeld & Chestin (1993) |
| 56 | Bulgaria | Temperate broadleaf & mixed forests | 41.6 | 24.6 | 5 | 3 | NA | 0.42 | 128 | Genov et al. (2008) |
| 57 | Bulgaria | Temperate broadleaf & mixed forests | 41.8 | 24.8 | 9 | 3 | 0.51 | 0.52 | 148 | Genov et al. (2010) |
| 58 | Estonia | Temperate broadleaf & mixed forests | 59.0 | 26.0 | 4 | 2 | 0.23 | 0.35 | 142 | Vulla et al. (2009) |
| 59 | Norway | Boreal forests & taiga | 69.0 | 28.0 | 4 | 2 | 0.27 | 0.34 | 137 | Persson et al. (2001) |
| 60 | Belarus | Temperate broadleaf & mixed forests | 55.5 | 30.0 | 1 | 2 | 0.19 | NA | 732 | Sidorovich (2006) |
| 61 | Russia | Boreal forests & taiga | 60.1 | 31.8 | 7 | 2 | 0.08 | NA | 640 | Novikov et al. (1969) |
| 62 | Russia | Boreal forests & taiga | 67.8 | 32.5 | 1 | 1 | 0.64 | NA | 250 | Semenov-Tian-Shanskii (1972) |
| 63 | Russia | Temperate broadleaf & mixed forests | 56.3 | 32.7 | 12 | 4 | 0.59 | 0.6 | 474 | Ogurtsov (2018) |
| 64 | Russia | Temperate broadleaf & mixed forests | 56.5 | 33.0 | 1 | 1 | 0.05 | NA | 18 | Pazhetnov (1990) |
| 65 | Russia | Temperate broadleaf & mixed forests | 56.5 | 33.0 | 4 | 3 | 0.22 | NA | 200 | Pazhetnov (1990) |
| 66 | Russia | Boreal forests & taiga | 63.8 | 33.0 | 1 | 1 | 0.15 | NA | 114 | Vaisfeld & Chestin (1993) |
| 67 | Russia | Boreal forests & taiga | 58.5 | 37.8 | 7 | 2 | 0.35 | NA | 258 | Kalecki (1973) |
| 68 | Russia | Boreal forests & taiga | 58.6 | 38.0 | 7 | 2 | 0.15 | NA | 209 | Razumovsky (1966) |
| 69 | Russia | Temperate broadleaf & mixed forests | 43.8 | 40.4 | 1 | 1 | 0.32 | NA | 108 | Vaisfeld & Chestin (1993) |
| 70 | Turkey | Temperate broadleaf & mixed forests | 40.5 | 41.5 | 1 | 1 | 0.39 | NA | 72 | Ambarl (2015) |
| 71 | Russia | Temperate broadleaf & mixed forests | 53.1 | 56.5 | 1 | 1 | 0.28 | NA | 228 | Sharafutdinov & Korotkov (1974) |
| 72 | Russia | Temperate broadleaf & mixed forests | 53.0 | 57.1 | 1 | 1 | 0.08 | NA | 140 | Vaisfeld & Chestin (1993) |
| 73 | India | Temperate broadleaf & mixed forests | 32.0 | 76.0 | 5 | 4 | 0.11 | NA | 222 | Rathore & Chauhan (2014) |
| 74 | India | Temperate coniferous forests | 32.5 | 76.8 | 5 | 4 | 0.11 | NA | 222 | Rathore (2008) |
| 75 | Kazakhstan | Montane grasslands and shrublands | 45.0 | 80.3 | 9 | 5 | 0.37 | NA | 884 | Grachev & Fedosenko (1977) |
| 76 | Russia | Boreal forests & taiga | 64.0 | 88.0 | 4 | 3 | 0.14 | NA | 39 | Vaisfeld & Chestin (1993) |
| 77 | Russia | Temperate coniferous forests | 52.3 | 92.8 | 1 | 1 | 0.23 | NA | 1279 | Vaisfeld & Chestin (1993) |
| 78 | Mongolia | Deserts and xeric shrublands | 43.4 | 97.2 | 1 | 1 | NA | NA | 365 | Schaller et al. (1993) |
| 79 | Russia | Montane grasslands and shrublands | 51.7 | 100.6 | 1 | 1 | 0.11 | NA | 895 | Vaisfeld & Chestin (1993) |
| 80 | Japan | Temperate broadleaf & mixed forests | 41.9 | 140.3 | 2 | 2 | NA | 0.17 | 18 | Nomura & Higashi (2000) |
| 81 | Japan | Temperate broadleaf & mixed forests | 41.9 | 140.3 | 7 | 6 | 0.45 | 0.59 | 44 | Ohdachi & Aoi (1987) |
| 82 | Japan | Temperate broadleaf & mixed forests | 41.9 | 140.3 | 10 | 7 | 0.16 | 0.14 | 223 | Sato et al. (2005) |
| 83 | Japan | Temperate coniferous forests | 45.0 | 142.0 | 9 | 6 | 0.13 | 0.16 | 90 | Aoi (1985) |
| 84 | Japan | Temperate coniferous forests | 45.2 | 142.0 | 4 | 3 | 0.14 | 0.31 | 73 | Ohdachi & Aoi (1987) |
| 85 | Japan | Temperate coniferous forests | 42.7 | 142.7 | 9 | 6 | 0.24 | 0.32 | 115 | Sato et al. (2005) |
| 86 | Japan | Temperate coniferous forests | 43.7 | 142.9 | 1 | 1 | 0.06 | 0.06 | 23 | Ohdachi & Aoi (1987) |
| 87 | Japan | Temperate coniferous forests | 43.7 | 142.9 | 7 | 6 | 0.13 | 0.12 | 218 | Sato et al. (2005) |
| 88 | Japan | Temperate broadleaf & mixed forests | 42.8 | 143.7 | 4 | 4 | 0.13 | 0.18 | 117 | Sato et al. (2004) |
| 89 | Japan | Temperate broadleaf & mixed forests | 42.8 | 143.7 | 6 | 4 | 0.23 | 0.19 | 34 | Sato et al. (2004) |
| 90 | Japan | Temperate coniferous forests | 44.1 | 145.2 | 8 | 5 | 0.14 | NA | 128 | Ohdachi & Aoi (1987) |
| 91 | Russia | Boreal forests & taiga | 60.0 | 148.0 | 11 | 3 | 0.47 | NA | 565 | Chernjavskij & Petrichenko (1984) |
| 92 | Russia | Boreal forests & taiga | 66.0 | 159.1 | 12 | 4 | 0.28 | NA | 1044 | Chernjavskij & Petrichenko (1984) |
| 93 | Russia | Boreal forests & taiga | 65.8 | 159.8 | 6 | 3 | 0.34 | NA | 108 | Krechmar (1995) |
| 94 | Russia | Tundra | 65.6 | 169.1 | 4 | 2 | 0.28 | NA | 107 | Krechmar (1995) |
| 95 | Russia | Tundra | 67.3 | 170.4 | 3 | 2 | 0.13 | NA | 148 | Krechmar (1995) |
| 96 | Russia | Tundra | 65.1 | 170.6 | 4 | 2 | 0.17 | NA | 112 | Krechmar (1995) |

**Appendix S7**. References (n=70) from which data on brown bear diet across its distribution range, including 96 study areas, were gathered. The ID of the study areas of each publication is also provided.

| **ID Study areas** | **Reference** |
| --- | --- |
| 70 | Ambarl, H. Litter size and basic diet of brown bears (*Ursus arctos*, Carnivora) in northeastern Turkey. *Mammalia* **80,** 235–240 (2015). |
| 83 | Aoi, T. Seasonal change in food habits of Ezo brown bear (*Ursus arctos yesoensis*) in Northern Hokkaido. *Research Bulletins of the College Experiment Forests Hokkaido University* **42,** 721–732 (1985). |
| 20 | Aune, K. E. *Rocky Mountain Front Grizzly Bear Monitoring and Investigation* (Department of Fish, Wildlife and Parks. Bureau of Land Management, 1985). |
| 19 | Aune, K. E. Comparative ecology of black and grizzly bears on the Rocky Mountain Front, Montana. *International Conference on Bear Research and Management* **9**, 365–374 (1994). |
| 18 | Aune, K. E. & Kasworm, W. *Final Report East Front Grizzly Bear Study* (Department of Fish, Wildlife and Parks, 1989). |
| 33 | Berducou, C., Faliu, L. & Barrat, J. Le regime alimentaire de’lours brun des pyrenees, d'après l'analyse des laissées récoltées en 1977, 1978 et 1979. *Acta Zool. Fennica* **17**, 153–156 (1983). In French. |
| 32 | Braña, F., Naves, J. & Palomero, G. Hábitos alimenticios, configuración de la dieta del oso pardo en la Cordilla Cantábrica. *El Oso Pardo* (eds. Naves, J. & Palomero, G.). 81-104 (Ministerio de Medio Ambiente, 1993). In Spanish. |
| 1,3 | Chernjavskij, F. & Krechmar, M. A. Brown bear in Chukotka tundra. Bears of Russia and adjacent countries - state of populations, vol. 2. *Proceedings Of The 6th Conference Of Specialists, Studying Bears, Central Forest Reserve, Tver Oblast, Russia* (eds. Chestin, I. E. & Uspensky, S. M.). 75–88 (Central Forest State Reserve of The Ministry of Environmental Protection, 1993). |
| 91, 92 | Chernjavskij, F. & Petrichenko, V. Diet of brown bears in north-eastern Siberia. *Moscow Univ. Biol. Sci. Bull* **89,** 33–41 (1984). |
| 44 | Cicnjak, L., Huber, D., Roth, H. U., Ruff, R. L. & Vinovrski, Z. Food habits of brown bears in Plitvice Lakes National Park, Yugoslavia. *Bears: Their Biology and Management* **7**, 221–226 (1987). |
| 36 | Ciucci, P., Tosoni, E., Di Domenico, G., Quattrociocchi, F. & Boitani, L. Seasonal and annual variation in the food habits of Apennine brown bears, central Italy. *J. Mammal* **95**, 572–586 (2014). |
| 30 | Clevenger, A. P., Purroy, F. J. & Pelton, M. R. Food habits of brown bears (*Ursus arctos*) in the Cantabrian Mountains, Spain. *J. Mammal* **73**, 415 – 421 (1992). |
| 27 | Craighead, J. J., Summer, J. S. & Mitchell, J. A. *The Grizzly Bears of Yellowstone: Their Ecology In The Yellowstone Ecosystem 1959-1992* (Island Press, 1995). |
| 13 | Cristescu, B., Stenhouse, G. B. & Boyce, M. S. Grizzly bear response to open-pit mining in Western Alberta, Canada. *Glob. Ecol. Conserv.* **4**, 207–220 (2015). |
| 39, 40 | Dahle, B., Sørensen, O., Wedul, E., Swenson, J. E. & Sandegren, F. The diet of brown bears *Ursus arctos* in central Scandinavia: effect of access to free-ranging domestic sheep Ovis aries. *Wildlife Biol.* **4**, 147–158 (1998). |
| 37 | Di Domenico, G., Tosoni, E., Boitani, L. & Ciucci, P. Efficiency of scat-analysis lab procedures for bear dietary studies: The case of the Apennine brown bear. *Mamm. Biol.* **77**, 190–195 (2012). |
| 35 | Elgmork, K. & Kaasa, J. Food habits and foraging of the brown bear *Ursus arctos* in central South Norway. *Ecography* **15**, 101–110 (1992). |
| 29 | Gau, R. J., Case, R., Penner, D. F. & McLoughlin, P. D. Feeding patterns of barren-ground grizzly bears in the Central Canadian Arctic. *Arctic* **55**, 339–344 (2002). |
| 56 | Genov, P., Dimitrova, D., Georgiev, T., Draganov, V., Banchev, P., Arabadjiev, D. & Mirchev, R. Studies on bear and wolf in the game breeding station “Shiroka poljana” with a view to their management. *Faculty of Natural Sciences* **18**, 173-189 (2008). |
| 57 | Genov, P., Dzhindzhieva, A. & Bedrov, G. Feeding of the brown bear (*Ursus arctos*) in the game breeding station "Kormisosh", West Rhodopes in *Anniversary Scientific Conference “Bulgaria and Bulgarians in Europe”* 601-608 (Union of Scientists in Bulgaria, 2010). |
| 75 | Grachev, Y. & Fedosenko, A. *Ursus arctos* in Djungarsky Alatau. *Zoologicheskiy Zhurnal* **66**, 120–129 (1977). |
| 42 | Große, C., Kaczensky, P. & Knauer, F. Ants: a food source sought by Slovenian brown bears (*Ursus arctos*)? *Can. J. Zool.* **81**, 1996–2005 (2003). |
| 15 | Hamer, D. & Herrero, S. Grizzly bear food and habitat in the Front Ranges of Banff National Park, Alberta. *International Conference on Bear Research and Management* **7**, 199–213 (1987). |
| 12 | Hamilton, A. & Bunnell, F. Foraging strategies of coastal grizzly bears in the Kimsquit river valley, British Columbia. *International Conference on Bear Research and Management* **7**, 187–197 (1987). |
| 47 | Jamnicky, J. The food of the brown bear (*Ursus arctos* L.) in the Tatra region. *Folia Venatoria* **18**, 197–213 (1988). |
| 67 | Kalecki, M. L. On the ecology of brown bears in Darvin Reserve. *Natural Resources of Mologa-Sheksna Lowland: Terrestrial Vertebrates* (ed. General Department of Hunting and Reserves.The Council of Ministers of the RSFSR State Reserve Darwin) 13–40 (North-West Book Publishing, 1973). |
| 41 | Kavcic, I., Adamic, M., Kaczensky, P., Krofel, M., Kobal, M. & Jerina, K. Fast food bears: brown bear diet in a human-dominated landscape with intensive supplemental feeding. *Wildlife Biol.* **21**, 1–8 (2015). |
| 25 | Knight, R. R., Blanchard, B. M. & Kendall, K. C. *Yellowstone Grizzly Bears Investigations* (Annual report of the Interagency Grizzly Bear Study Team, 1982). |
| 49 | Frackowiak, W. & Gula, R. The autumn and spring diet of brown bear *Ursus arctos* in the Bieszczady Mountains of Poland. *Acta theriologica* **37**, 339–344 (1992). |
| 93, 94, 95, 96 | Krechmar, M. A. Geographical aspects of the feeding of the brown bear (*Ursus arctos* L.) in the extreme Northeast of Siberia. *Russ. J. Ecol* **26**, 436–443 (1995). |
| 34 | Lagalisse, Y. *Etude Coproscpique Du Regime Alimentaire D'une Population D'ours Bruns (Ursus Arctos) Reintroduite Dans Les Pyrenees (1996-1999).* PhD Thesis (l’Universite Paul-Sabatier de Toulouse, 2002). In French. |
| 16,21,23,24 | Mace, R. D. & Jonkel, C. J. Local food habits of the grizzly bear in Montana. *Bears: their biology and management* **6**, 105–110 (1986). |
| 10 | MacHutchon, A.G. & Wellwood, D.W. Grizzly bear food habits in the northern Yukon, Canada. *Ursus* **14**, 225–235 (2003). |
| 26 | Mattson, D. J., Blanchard, B. M. & Knight, R. R. Food habits of Yellowstone grizzly bears, 1977–1987. *Can. J. Zool.* **69**, 1619–1629 (1991). |
| 17 | McLellan, B. N. & Hovey, F. W. The diet of grizzly bears in the Flathead River drainage of southeastern British Columbia. *Can. J. Zool.* **73**, 704–712 (1995). |
| 28 | Mealey, S. P. The natural food habits of grizzly bears in Yellowstone National Park, 1973-74*. Bears: their biology and management* **4**, 281–292 (1980). |
| 50 | Mertzanis, G. Brown bear in Greece: distribution, present status - Ecology of a northern Pindus sub-population. *International Conference on Bear Research and Management* **14***,* 249–270 (1992). |
| 48 | Mertzanis, Y., Bousdouras, D. & Bourdakis, S. Status of brown bear (*Ursus arctos* L.) populations and habitat in the area of Prespa lakes in *International Symposium Sustainable Development of Prespa Region,* 55–64 (2000). |
| 14 | Munro, R. H. M., Nielsen, S. E., Price, M. H., Stenhouse, G. B. & Boyce, M. S. Seasonal and diel patterns of grizzly bear diet and activity in West-Central Alberta. *J. Mammal.* **87**, 1112–1121 (2006). |
| 6 | Murie, A. *The Grizzlies of Mount McKinley* (National Park Service Scientific Monographs, 1981). |
| 31 | Naves, J., Fernández-Gil, A., Rodríguez, C. & Delibes, M. Brown bear food habits at the border of its range: a long-term study. *J. Mammal.* **87**, 899–908 (2006). |
| 80 | Nomura, F. & Higashi, S. Effects of food distribution on the habitat usage of a female brown bear Ursus arctos yesoensis in a beech-forest zone of northernmost Japan. *Ecol. Res* **1**, 209–217 (2000). |
| 61 | Novikov, G. A., Airapetjants, A. E., Pukinsky, Y. B., Timofeeva, E. K. & Fokin, I. M. Some peculiarities of population of brown bears in the Leningrad district. *Zoologicheskii Zhurnal* **68**, 885–900 (1969). |
| 63 | Ogurtsov, S. S. The diet of the brown bear (*Ursus arctos*) in the Central Forest Nature Reserve (Russia), based on scat analysis data. *Zoologicheskii zhurnal* **97**, 486–502 (2018). |
| 81, 84, 86, 90 | Ohdachi, S. & Aoi, T. (1987) Food habits of brown bears in Hokkaido, Japan. *International Conference on Bear Research and Management* **7**, 215–220 (1987). |
| 51 | Paralikidis, N. P., Papageorgiou, N. K., Kontsiotis, V. J. & Tsiompanoudis, A. C. The dietary habits of the brown bear (*Ursus arctos*) in western Greece. *Mammalian Biology - Zeitschrift für Säugetierkunde* **75**, 29–35 (2010). |
| 64, 65 | Pazhetnov, V. S. *Brown bear* (Agropromizdat, 1990). In Russian. |
| 11 | Pearson, A. M. *The Northern Interior grizzly bear* *Ursus arctos* L. (Environmental Canada - Wildlife Service, 1975). |
| 59 | Persson, I. L., Wikan, S., Swenson, J. E. & Mysterud, I. The diet of the brown bear *Ursus arctos* in the Pasvik Valley, northeastern Norway. *Wildlife Biol.* **7**, 27–37 (2001). |
| 9 | Phillips, M. K. Behavior and habitat use of grizzly bears in northeastern Alaska. *Bears: Their Biology and Management* **7**, 159–176 (1987). |
| 73 | Rathore, B. C. & Chauhan, N. P. S. The food habits of the Himalayan brown bear *Ursus arctos* (Mammalia: Carnivora: Ursidae) in Kugti Wildlife Sanctuary, Himachal Pradesh, India. *J. Threat. Taxa* **6**, 6649–6658 (2014). |
| 68 | Razumovsky, B. I. Bears (*Ursus arctos arctos* L.) of the Mologa-Sheksna watershed. *Zoologicheskii Zhurnal* **45**, 725–729 (1966). |
| 5 | Reynolds, H. V. *North Slope Grizzly Bear Studies* (Department of Fish and Game,1980). |
| 46 | Rigg, R. & Gorman, M. Diet of brown bears (*Ursus arctos*): New results from Tatras region and a comparison of research methods. *Vysskum A Ochrana Cicavcov Na Slovensku* **7**, 61–79 (2005). |
| 82, 85, 87 | Sato, Y., Mano, T. & Takatsuki, S. Stomach contents of brown bears Ursus arctos in Hokkaido, Japan. *Wildlife Biol.* **11**, 133–144 (2005). |
| 88,89 | Sato, Y., Aoi, T., Kaji, K. & Takatsuki, S. Temporal changes in the population density and diet of brown bears in eastern Hokkaido, Japan. *Mammal Study* **29**, 47–53 (2004). |
| 78 | Schaller, G., Tulgat, R. & Navantsatsvalt, B. Observations on the Gobi brown bear in Mongolia. Bears of Russia and adjacent countries - state of populations*,* vol. 2. *Proceedings of The 6th Conference Of Specialists, Studying Bears, Central Forest Reserve, Tver Oblast, Russia* (eds. Chestin, I. E. & Uspensky, S. M.). 110–122 (Central Forest State Reserve of The Ministry of Environmental Protection, 1993). |
| 22 | Servheen, C. Grizzly bear food habits, movements, and habitat selection in the Mission Mountains, Montana. *J. Wildl. Manage.* **47**, 1026–1035 (1983). |
| 62 | Semenov-Tian-Shanskii, C. The brown bear in the Lapland Reserve, U.S.S.R. *Aquilo Serie Zoologica* **13**, 98–102 (1972). |
| 71 | Sharafutdinov, I. & Korotkov, A. On the ecology of the brown bear in the southern Urals. *International Conference on Bear Research and Management* **3**, 309–311 (1974). |
| 45 | Skuban, M., Find´o, S. & Kajba, M. Human impacts on bear feeding habits and habitat selection in the Polana Mountains, Slovakia. *Eur. J. Wildl. Res.* **62**, 353–364 (2016). |
| 7,8 | Stelmock, J. & Dean, F. Brown bear activity and habitat use, Denali National Park - 1980. *International Conference on Bear Research and Management* **6**, 155–167 (1986). |
| 53 | Stofik, J., Merganic, J., Merganicová, K. & Saniga, M. Seasonal changes in food composition of the brown bear (*Ursus arctos*) from the edge of its occurrence – Eastern Carpathians (Slovakia). *Folia Zool.* **62**, 222–231 (2013). |
| 60 | Sidorovich, V. Ecological studies on brown bear (*Ursus arctos*) in Belarus: distribution, population trends and dietary structure. *Acta Zoologica Lituanica* **16**, 185–190 (2006). |
| 43 | Stenset, N.E. *et al*. Seasonal and annual variation in the diet of brown bears *Ursus arctos* in the Boreal forests of southcentral Sweden. *Wildl. Biol.* **22**, 107–116 (2016) |
| 55, 66, 69, 72, 76, 77, 79 | Vaisfeld, M. A. & Chestin, I. E. (Eds). *Game Animals of Russia and Adjacent Countries and Their Environment: Bears - Brown Bear, Polar Bear, Asian Black Bear*. Nauka, 1993. |
| 52 | Vlachos, C. G., Dimitriou, M., Kritikou, K., Chouvardas, D. & Bakaloudis, D. E. Seasonal food habits of the European brown bear (*Ursus arctos*) in the Pindos Mountains, Western Greece. *Folia Zool.* **49**, 19 – 25 (2000). |
| 58 | Vulla, E. *et al.* Carnivory is positively correlated with latitude among omnivorous mammals: evidence from brown bears, badgers and pine martens. *Ann. Zool. Fenn.* **46**, 395–415 (2009). |
| 38 | Zunino, F. & Herrero, S. The status of the brown bear (*Ursus arctos*) in Abruzzo National Park, Italy, 1971. *Biol. Conserv.* **4**, 263–272 (1972). |
